# Accent modulates access to word meaning: Evidence for a speaker-model account of spoken word recognition

**DOI:** 10.1016/j.cogpsych.2017.08.003

**Published:** 2017-09-04

**Authors:** Zhenguang G. Cai, Rebecca A. Gilbert, Matthew H. Davis, M. Gareth Gaskell, Lauren Farrar, Sarah Adler, Jennifer M. Rodd

**Affiliations:** a University College London, United Kingdom; b University of East Anglia, United Kingdom; c MRC Cognition & Brain Sciences Unit, Cambridge, United Kingdom; d University of York, United Kingdom

**Keywords:** Spoken word recognition, Semantic ambiguity, Accent, Dialect

## Abstract

Speech carries accent information relevant to determining the speaker’s linguistic and social background. A series of web-based experiments demonstrate that accent cues can modulate access to word meaning. In Experiments 1–3, British participants were more likely to retrieve the American dominant meaning (e.g., *hat* meaning of “bonnet”) in a word association task if they heard the words in an American than a British accent. In addition, results from a speeded semantic decision task (Experiment 4) and sentence comprehension task (Experiment 5) confirm that accent modulates on-line meaning retrieval such that comprehension of ambiguous words is easier when the relevant word meaning is dominant in the speaker’s dialect. Critically, neutral-accent speech items, created by morphing British- and American-accented recordings, were interpreted in a similar way to accented words when embedded in a context of accented words (Experiment 2). This finding indicates that listeners do not use accent to guide meaning retrieval on a word-by-word basis; instead they use accent information to determine the dialectic identity of a speaker and then use their experience of that dialect to guide meaning access for all words spoken by that person. These results motivate a speaker-model account of spoken word recognition in which comprehenders determine key characteristics of their interlocutor and use this knowledge to guide word meaning access.

## 1 Introduction

Successful language comprehension requires that listeners rapidly and accurately retrieve the meanings that speakers intend to convey in their speech. While meaning retrieval can be quite straightforward when the listener and the speaker have a single or preferred meaning for a word, the process of retrieving the speaker-intended meaning is made more challenging when the listener and the speaker have different preferred meanings for the word, as in the case of words which have different preferred or dominant meanings in different varieties of a language (e.g., “bonnet” typically refers to a car part in British English but a type of hat in American English). In this case, one useful cue for the listener is the accent of the speaker, which can be used to infer the speaker’s dialectic background and in turn the likely intended meaning of cross-dialectically ambiguous words. The study assesses whether listeners make use of speaker accent in accessing word meanings, and if so, what the mechanism is that supports such accent-based meaning inference.

To quote George Bernard Shaw: “*England and America are two nations divided by a common language*”.^1^ Despite being (mostly) mutually intelligible, British and American English differ systematically in the accent in which words are pronounced. Furthermore, the meanings of common everyday words often differ between the two dialects (we use the term dialect to refer to a variety of a language, e.g., British English, and the term accent to refer more specifically to the manner of speech in a dialect; in addition, we use *British accent* and *American accent* respectively as an umbrella term for the set of different regional accents within the United Kingdom and the United States). While an American would say they live in an “apartment”, a British person would call their single-story residence a “flat”, a word that could also mean a deflated tire or the shape of a pancake. Comparable differences of accent and word meaning arise between many pairs of geologically-separate language communities (e.g., South American and European Spanish or Portuguese) and sometimes even between different dialects of the same language community (e.g., the north-south distinction between the use of “tea” and “dinner” to refer to the evening meal in England). Exposure to these variations in speaker accent and meaning is increasingly the norm rather than the exception in daily communication as a result of immigration and long-distance communication across different dialectic isoglosses (e.g., between the United Kingdom and the United States) and exposure to international media (e.g., TV programmes). Successful communication between individuals from different language communities may require that listeners use many available cues, speaker accent for instance, to determine what words and meanings any given speaker is likely to convey.

Speaker accent does not lead to random variation in the speech sounds but arises due to systematic variation in the phonetic realisations of words among different dialects. Several studies have shown that listeners experience difficulty in identifying words that are spoken in an accent different from their own (e.g., Floccia, Goslin, Girard, & Konopczynski, 2006). However, it is currently unclear whether the accent in which words are spoken also impacts meaning access. The paucity of such research probably reflects an assumption that accent variations change the surface form of speech but not the underlying words or meanings conveyed. The word “tree” has the same meaning regardless whether it is spoken by a British or American English speaker (i.e. heard in a British or American accent). Such an assumption, however, is unjustified due to dialect-induced changes in the meanings typically conveyed by different words. For instance, the word “bonnet” more often means a car part than a hat in British English, but the reverse is true in American English (American English speakers use the word “hood” for the equivalent car part).

Words with different meanings in different dialects provide an ideal case for testing whether accent information can directly influence meaning access (by which we mean the on-line retrieval of a stored lexical semantic representation for a word). When listeners who are sufficiently familiar with both British and American English hear the word “bonnet”, they are likely to activate to some extent both *car-part* and *hat* meanings (e.g., Swinney, 1979). One of the meanings will then be quickly selected based on evidence derived from various sources (e.g., meaning frequency and prior context; Duffy, Morris, & Rayner, 1988). If speaker accent modulates meaning access, the hat meaning of "bonnet" should then be selected with a greater likelihood if the word is spoken in an American than British accent. The present study thus investigates whether, and how, listeners use speaker accent in retrieving the speaker’s intended meaning, using cross-dialectically ambiguous words like “bonnet”.

In motivating the experiments presented in this paper, we begin by reviewing the literature on semantic ambiguity resolution before discussing existing evidence that surface features of speech can influence meaning access for words and sentences. We then discuss and contrast two possible mechanisms via which surface speech forms can affect meaning access. We first show, in a web-based experiment, that listeners use accent cues to guide retrieval of dialect-specific dominant meanings of ambiguous words (e.g., changing the likelihood of retrieving the *hat* or *car-part* meaning of “bonnet” when heard in an American or British accent). We then report two follow-up experiments to further determine the underlying processing mechanism by which accent modulates access to word meanings. Finally, two experiments using speeded tasks (semantic relatedness judgement and sentence interpretation) both show that speaker accent modulates on-line processes underlying rapid meaning access and sentence comprehension. The results of these five experiments suggest that current accounts of spoken word recognition and meaning access need to be extended to explain how listeners use accent cues to infer the likely meanings of spoken words. We finish the paper by describing a speaker-model account of spoken word recognition and meaning retrieval that can accommodate these findings.

### 1.1 Lexical ambiguity resolution in language comprehension

Research on lexical ambiguity resolution has shown that comprehenders are extremely sensitive to contextual and distributional factors when selecting a meaning for a semantically ambiguous word such as “bark” (Cai & Vigliocco, in press; Twilley & Dixon, 2000; Vitello & Rodd, 2015). The semantic cues present in preceding words play a key role in the disambiguation of ambiguous words within sentences. Comprehenders are also sensitive to the relative frequency with which different word meanings are used in the language. This is apparent in the greater ease with which more frequent (dominant) meanings are accessed compared to lower-frequency (subordinate) meanings. For example, when an ambiguous word such as “bark” occurs in a constraining sentence context (e.g., “The tree/dog had an unusual BARK”), reading times are longer when the lower frequency tree-related meaning is used (e.g., Duffy et al., 1988). In neutral contexts, readers or listeners assign the dominant meaning to ambiguous words which can result in effortful reinterpretation if subsequent linguistic input turns out to favour the subordinate meaning (e.g., “He noticed the BARK from the old TREE”; e.g., Rayner & Duffy, 1986; Rodd, Johnsrude, & Davis, 2010). In a series of recent studies using ambiguous words, Rodd and colleagues (Rodd, Cutrin, Kirsch, Millar, & Davis, 2013; Rodd et al., 2016) have shown that listeners are also sensitive to how recently different word meanings have been used. Exposure to particular meanings for ambiguous words leads to changes in dominance across a range of timescales from minutes to days and years. These effects have been studied using a word association task in which participants generate their responses to ambiguous words presented in isolation (e.g., “bark”), without a sentence context. Prior exposure to an ambiguous word which the sentence context disambiguated towards its subordinate meaning (e.g., “The tree had an unusual BARK ”) tens of minutes earlier led to a considerable increase in responses associated with that subordinate meaning (Rodd et al., 2013). Such word-meaning priming indicates that prior exposure modulates the availability of word meanings. In follow-up work Rodd et al. (2016) showed that repeated and extensive exposure to specific meanings (e.g., in the context of individuals’ hobbies) leads to substantial inter-individual differences in word association responses that reflect underlying differences in their experiences with these words both within the current day and over several years. Poort, Warren, and Rodd (2016) have shown that the effect of prior meaning exposure also occurs across languages, for example when a written word like “room” exists both in English and in Dutch but with different meanings.

Existing research has further shown that the tone of voice or emotion in which a word is spoken can bias lexical disambiguation of homophones that have one affective (happy or sad) meaning (e.g., “flower-flour”, “die-dye”). Nygaard and Lunders (2002) found that participants were more likely to assign the affective meaning to these words if they were spoken in a congruent emotional tone compared with a neutral tone. On the basis of these findings, it may be expected that listeners will utilise accent cues when interpreting words that have different meaning dominances in different dialects. However, the influence of emotional tone on meaning access could arise from a simpler mechanism than that involved in processing accent variation. There is a straightforward semantic relationship between the semantic category that is associated with the tone-of-voice (e.g., *sadness*) and the meaning that is favoured (i.e. *sad* meanings). Hearing a sad voice might thus produce direct semantic priming for a cluster of sadness-related meanings. In contrast, in the case of accent, there is no reason to think that hearing a British or American accent would favour clusters of *car-part* or *hat* meanings per se; only when these accents accompany a word that has different dominant meanings for different dialects (e.g., “bonnet”), would we expect an effect of accent on meaning access. This might require a lexically specific inference concerning the meaning intended by speakers with different accents. The goal of this paper is to explore whether and how this form of inference operates.

Many authors have argued that successful communication requires that interlocutors constantly attend to each other’s state of mind in a way that might support speaker- or interlocutor-specific inferences (e.g., Pickering & Garrod, 2004; Sumner, Kim, King, & McGowan, 2014). For instance, in dialogue, listeners use their knowledge of which objects their interlocutor can see in order to identify potential referents (e.g., Hanna, Tanenhaus, & Trueswell, 2003; Keysar, Barr, Balin, & Brauner, 2000). Indeed, more recent research has suggested that listeners can derive a model of the speaker from speech input alone (Tesink et al., 2009; Van Berkum, Van den Brink, Tesink, Kos, & Hagoort, 2008). For instance, listeners detect a semantic anomaly at hearing “If only I looked like Britney Spears” spoken by a male voice, or hearing “I have a large tattoo on my back” spoken in an upper-class accent (Van Berkum et al., 2008). Martin, Garcia, Potter, Melinger, and Costa (2016) also showed that listeners are sensitive to lexical choices by the speaker, given the speaker’s accent: listeners had difficulty (as reflected in ERPs) understanding a more British expression (e.g., use of “holiday” to mean a period of leisure) in a sentence spoken in an American accent and a more American expression (e.g., “vacation”) in a sentence spoken in a British accent. These findings suggest that listeners have expectations of what message and lexical expressions speakers of a particular gender, social class or nationality are likely to produce.

On these grounds, then, it seems reasonable to expect that listeners can employ speaker accent (and their past knowledge of words and meanings that they have heard in that accent) to interpret words that have different meaning dominances between dialects. However, this leaves unclear the specific mechanism by which accent could modulate meaning access.

### 1.2 Mechanisms for accent processing in spoken word recognition

There are two theoretically distinct ways in which variation in a speaker’s accent is treated by models of spoken word recognition. First, surface phonetic details of spoken words may be retained in the lexicon and used in identification (i.e. episodic accounts, as initially proposed by Goldinger, 1996; for reviews see Luce & McLennan, 2005; Samuel, 2011). Alternatively, surface details may be mapped onto abstract wordform representations devoid of surface details such as accent, speech rate and loudness (i.e. abstractionist accounts; see Bowers, 2000; McQueen, Cutler, & Norris, 2006 for discussion). Both of these approaches seek to explain how it is that spoken word recognition achieves impressive robustness despite variation in the surface form of words produced by speakers with different accents. In this section we will describe how episodic and abstractionist approaches as instantiated in current models might accommodate effects of accent on meaning access. Our goal in this paper is not to select between these types of account so much as to use them as a framework to guide our experimental investigations.

Early research on speech perception treated surface details caused by talker variability as noise to be normalised during word recognition and not retained within lexical representations of spoken words (e.g., Gerstman, 1968; Joos, 1948; Liberman, 1973; Verbrugge, Strange, Shankweiler, & Edman, 1976). However, awareness of the challenges caused by surface acoustic variation in speech led to computational models – such as the TRACE model (McClelland & Elman, 1986) – that include mechanisms for competitive selection in pre-lexical and lexical representations such that varied surface forms can be recognised as instances of a single abstract wordform. Importantly, the abstract lexical representations in these models produce an informational ‘bottle-neck’ that prevents surface characteristics of individual spoken tokens such as accent or pitch from contributing to subsequent semantic processing (McClelland & Elman, 1986; Norris, 1994). Within these models, tokens of a word such as “bonnet” will activate the same wordform node regardless of the speaker’s accent. Current abstractionist models therefore limit the opportunity for there to be any effect of accent on meaning access.

In contrast, episodic accounts assume that specific surface details of speech are part of the wordform representation stored in the lexicon. These models were explicitly designed to explain experimental evidence that acoustic and phonetic properties of individual spoken words can influence recognition (e.g., Goldinger, 1996, 1998; Pufahl & Samuel, 2014). For instance, Goldinger (1998) proposed that wordform representations consist of multiple overlapping memory traces from previous exposures to different tokens of the word. This mechanism is invoked to explain experimental findings that listeners show sensitivity to indexical and other surface variations during spoken word recognition (Goldinger, 1996; González & McLennan, 2007; Mullennix, Pisoni, & Martin, 1989; Pufahl & Samuel, 2014; Remez, Fellowes, & Rubin, 1997; Sheffert, Pisoni, Fellowes, & Remez, 2002; for reviews see Luce & McLennan, 2005, and Samuel, 2011). With respect to effects of speaker accent on meaning access, by assuming that lexical representations retain details such as accent, these models can straight-forwardly allow for accent information to directly influence word-meaning access. For instance, past linguistic experience that British- and American-accented tokens of “bonnet” are respectively used to refer to a car part and a hat can lead to the formation of links from different accent details to different meanings. Such a surface-detail mechanism would thus allow accent details in a speech token to feed forward to influence meaning retrieval (e.g., an American-accented token of “bonnet” would activate past American-accented memory traces which in turn more strongly activate the hat meaning).

While abstractionist models (in their current form) do not predict that accent-related phonetic cues should directly impact word-meaning access, it is important to note that the abstractionist view should not be taken as suggesting that accent and other indexical information is completely ignored or discarded – only that it does not contribute to word recognition. Abstractionist (and indeed episodic) lexical models are entirely compatible with the view that listeners make use of a parallel speaker-identification mechanism outside the lexicon. This ‘speaker model’ allows listeners to use a variety of linguistic and paralinguistic cues to build up a representation of the speaker that contains information about, for example, their age, gender, voice identity (e.g., Belin, Fecteau, & Bedard, 2004) or social background (Sumner et al., 2014). The speaker model provides a mechanism by which listeners could take into account such properties when interpreting speech (Tesink et al., 2009; Van Berkum et al., 2008). A similar mechanism could also operate when processing accented speech. That is, a speaker model could be established early on during communication and used to guide meaning access for subsequent speech. For instance, when hearing an American English speaker, an established speaker model might then boost accent-congruent meanings (e.g., the hat meaning for “bonnet”). Importantly, this ‘speaker model’ proposes that the processing of speaker accent takes place outside the lexicon and therefore does not place any specific requirement on precisely what information must be retained within the lexicon.

We have outlined two ways in which sensitivity to speaker accent might influence the selection between meanings of ambiguous words. According to a surface-detail account, accent-related phonetic cues are stored *in the lexicon* and can directly modulate word-meaning access. Alternatively, in a speaker-model account, accent cues are processed *outside the lexicon* and influence word-meaning access via a separate ‘indexical’ pathway. These two mechanisms make different predictions concerning whether accent effects on meaning access would be influenced by the strength of the specific accent cues found in individual speech tokens. Under the speaker-model account the strength of accent information in an individual token associated with a particular (familiar) speaker will be irrelevant. Thus, the accent effect should be similar for a word that is phonetically very different when spoken in British vs. American English (e.g., “fall”) and one that is phonetically similar (e.g., “mate”). In contrast, under the surface-detail account the strength of the accent for a particular lexical item matters: a word such as “fall” would have more accent details stored in its lexical memory traces that can preferentially activate word-meaning representations congruent with that dialect. Thus, strongly accented words should lead to larger accent effects on word-meaning access than more weakly-accented words such as “mate”.

### 1.3 Overview of experiments

We conducted five experiments to investigate whether and how the accent in which a word is heard modulates access to word meanings. To this end, the first three experiments used a word association task, which has been extensively used to study the factors that influence how ambiguous words are processed (e.g., Rodd et al., 2013, 2016) since it allows participants to interpret isolated spoken words in an unconstrained manner while nonetheless requiring disambiguation. In the task, participants had to generate an associate of an ambiguous spoken word heard in a particular accent; these responses were then used to identify the retrieved meaning. The critical ambiguous words had different dominant meanings between British and American English (e.g., “bonnet”, “flat”, “gas”), such that participants’ responses in the word association task would indicate which meaning they retrieved (e.g., an associate such as “hat” to the target word “bonnet” clearly indicates that the *hat* meaning was retrieved) and hence whether meaning access is modulated by accent. To reduce participants’ awareness of the accent manipulation, we manipulated accent (British vs. American) between participants in all the experiments such that each participant was exposed to only one accent. Experiment 1 established the word association paradigm used in the first three experiments, and allowed us to determine whether accent influences meaning retrieval equivalently for British and American participants, who may differ in their relative levels of experience with the non-native accent. Given that the results demonstrated accent sensitivity, Experiments 2 and 3 then contrasted the two possible mechanisms of accent modulation (the speaker-model and the surface-detail account) in order to better understand *how* accent information is incorporated into the spoken word recognition and speech comprehension. As word association is an off-line task that may not reflect on-line meaning access, in Experiments 4 and 5, we used two on-line tasks (a speeded semantic relatedness task and a speeded sentence interpretation task) to show that speaker accent modulates on-line word meaning retrieval.

## 2 Experiment 1

### 2.1 Method

#### 2.1.1 Design

To explore whether listeners were sensitive to speaker accent in comprehending words with different dominant meanings between accents, this experiment adopted a 2 (accent: British vs. American) × 2 (group: British vs. American participants) design. Accent was manipulated between participants (i.e. each participant was only exposed to one of the two accents) to avoid making the accent contrast more salient. We also conducted a test of phonetic similarity between a word’s British and American accented tokens, in an attempt to examine whether any accent effect found would be stronger for words where the two accent variants are highly dissimilar (e.g., “fall,” “quarter”) compared to words that sound more similar in the two accents (e.g., “mate”, “vest”).

#### 2.1.2 Participants

Participants were recruited from Mechanical Turk (each paid $4) or Facebook (participants who completed the survey were entered into a prize draw). The British/American participants had registered themselves on Mechanical Turk as native speakers of British/American English (or reported themselves to be so in case of participants recruited from Facebook) and, at the time of testing, reported that they resided in the UK/USA and had never lived in the USA/UK for more than three months. Participants who failed to meet these criteria were excluded, leaving 57 British participants (32 and 25 respectively in the British and American accent; average age = 32.6, SD = 10.7; 39 females) and 63 American participants (31 and 32 respectively in the British and American accent condition; average age = 31.7, SD = 10.1; 30 females).

#### 2.1.3 Materials

We first selected words that have different dominant meanings in British English (*British meaning* henceforth) and American English (*American meaning* henceforth). To do this, we pre-tested 44 words that we expected to have different dominant meanings in British and American English. Another 32 British participants and 27 American participants (who conformed to the above participant criteria) took part in the pre-test for a prize draw. In each trial of the pre-test, participants saw a word, together with the definition of one of its two intended meanings, and decided how familiar they were with that meaning of the word on a 7-point scale (with 1 designating “completely unfamiliar” and 7 designating “highly familiar”). A participant rated both meanings of a word on different trials and all the trials were randomly presented (see Appendix A for the ratings). We adopted three criteria in screening the words. First, a word should have both meanings rated higher than 2 by both the British and the American participants; this was to ensure that both meanings were sufficiently familiar to both British and American participants. Second, if a meaning (e.g., the *car-part* meaning of “bonnet”) is more familiar than the other meaning (e.g., the *hat* meaning of “bonnet”) for British participants, the reverse should be true for American participants. That is, a word should have different dominant meanings for British and American participants. Third, for each word, the difference of differences in ratings between the two meanings in the two participant groups should be greater than 2 (e.g., for “bonnet”, the difference between the British and American meaning familiarity ratings was 5.4 − 4.2 = 1.2 for British participants and 1.9 − 4.9 = −3 for American participants, making the difference of differences as 4.2). Such an interaction score in rating was to ensure that a word had sufficiently distinct meaning dominances between the two dialects to reveal an accent modulation on meaning access if there is such an effect. These criteria left us with 22 ambiguous words (see Appendix A). Across the familiarity ratings for these selected words, there was a British meaning advantage for British English speakers (British meanings: *M* = 6.41, *SD* = 0.43, range = 4.91–6.91; American meanings: *M* = 4.50, *SD* = 1.03, range = 3.03–6.38), and an American meaning advantage for American English speakers (British meanings: *M* = 4.26, SD = 1.37, range = 2.12–6.41; American meanings: *M* = 6.20, *SD* = 0.64, range = 4.76–7.00). An additional 22 relatively unambiguous words taken from Vitello (2014) were included as filler items. All words were recorded in a sound-proof booth, spoken by a female speaker of Southern British English and a female speaker of American English (from the northwest of the United States) of similar ages (mid 20s) and educational backgrounds.

#### 2.1.4 Procedure

The experiment was conducted using the online survey system Qualtrics. After giving informed consent, participants were randomly assigned to either the British or American accent condition. The survey started with a word-association task, followed by a meaning-clarification task, and then by a post-experiment questionnaire. In word association, participants heard a word (which could be repeated on demand) and then typed a word or phrase representing the first thing that came to their mind. There was no time limit, though participants were instructed to respond as quickly as possible. Trials (22 targets and 22 fillers) were presented in a random order. In meaning clarification, participants coded which meaning of the target word their response related to. We opted to have participants code their own responses as an unpublished experiment in our lab comparing coding by participants and by the experimenter showed 95% consistency between the two, suggesting that participant self-clarification is a very reliable method (indeed our results below showed that none of the participants randomly coded their responses). In addition, this approach avoids the potential bias that could be introduced from response coding by (UK-based) experimenters who may be less skilled at correctly interpreting the intended meanings of responses of American participants. In each trial, participants were presented with a word that they had heard in the word-association task (e.g., “bonnet”) together with the word-association response that they had given (e.g., “hat”). They clicked on one of the four choices that best described the meaning that their response related to. The first two were the definitions of the American meaning (e.g., “a woman’s or child’s hat tied under the chin”) and the British meaning (e.g., “the hinged metal canopy covering the engine of a motor vehicle”), with their order counter-balanced across items. Participants could also indicate that their response related to a meaning of that word other than the ones listed (Choice 3) or was due to mishearing the word (Choice 4). The meaning clarification task included all the 22 target ambiguous words and also 8 of the unambiguous filler words; these filler words were also included as a check for random responding. For filler words, the correct definition of the word’s meaning and an incorrect unrelated definition were listed as Choice 1 and 2, with their order counter-balanced across the filler items, in addition to Choice 3 (other meaning) and Choice 4 (mishearing). In the post-experiment questionnaire, we collected a participant’s demographic information (e.g., gender, age, first language). In particular, we asked participants whether they had lived in another English-speaking country for more than 3 months and which country if they had, and how often they had encountered American English (for British participants) or British English (for American participants) in their daily life on a four point scale, with 1–4 respectively denoting “less than once a month”, “once a month”, “once a week”, and “once a day”.

#### 2.1.5 Phonetic similarity rating

We recruited another 36 British participants via Facebook and other online resources and another 36 American participants from Mechanical Turk to rate the phonetic similarity between the British- and American-English pronunciations of each of the 22 target words. Seven filler words served as practice items. Two versions of materials were created: in one, participants first heard the British pronunciation of a word then its American-English pronunciation; in the other version the order was reversed. Participants rated how (dis)similar the two pronunciations were on a 7-point scale, with 7 denoting very dissimilar. The phonetic similarity ratings (see Appendix A) by British and American speakers were highly correlated (*r* = 0.909, *p* < 0.001), suggesting that speakers of the two dialects agreed on the strength of phonetic similarity between a word’s accent tokens.

### 2.2 Results

We first determined whether any participant had responded randomly in the meaning clarification task. If they had, we would expect the correct and incorrect definitions to be chosen with equal likelihoods. Out of the correct and incorrect definitions for the filler items, the correct definition was chosen 100% of the time for the majority of participants; for the remaining 4 participants, the correct definition was chosen more than 80% of the time. These results suggest that none of the participants had randomly responded in the meaning clarification of their responses and hence were all included in the analyses.

For target trials, a response was coded as British-meaning if it was clarified as relating to the dominant meaning in British English (e.g., the *car-*part meaning of “bonnet”), American-meaning if it was clarified as relating to the dominant meaning in American English (e.g., the *hat* meaning of “bonnet”), or an “other” response if the response was clarified as not relating to either given meaning or to be the result of mishearing. There were 2110 British- and American-meaning target responses and 530 “other” responses. An analysis comparing “other” responses with target responses revealed a main effect of participant group, with more “other” responses by the American than British participants (24% vs. 16% out of all responses; *β* = 0.67, *SE* = 0.30, *z* = 2.27, *p* = 0.024); critically there was no significant effects of accent or interaction (|*z*|s < 1, *p*s > 0.10).

**Fig. 1:**
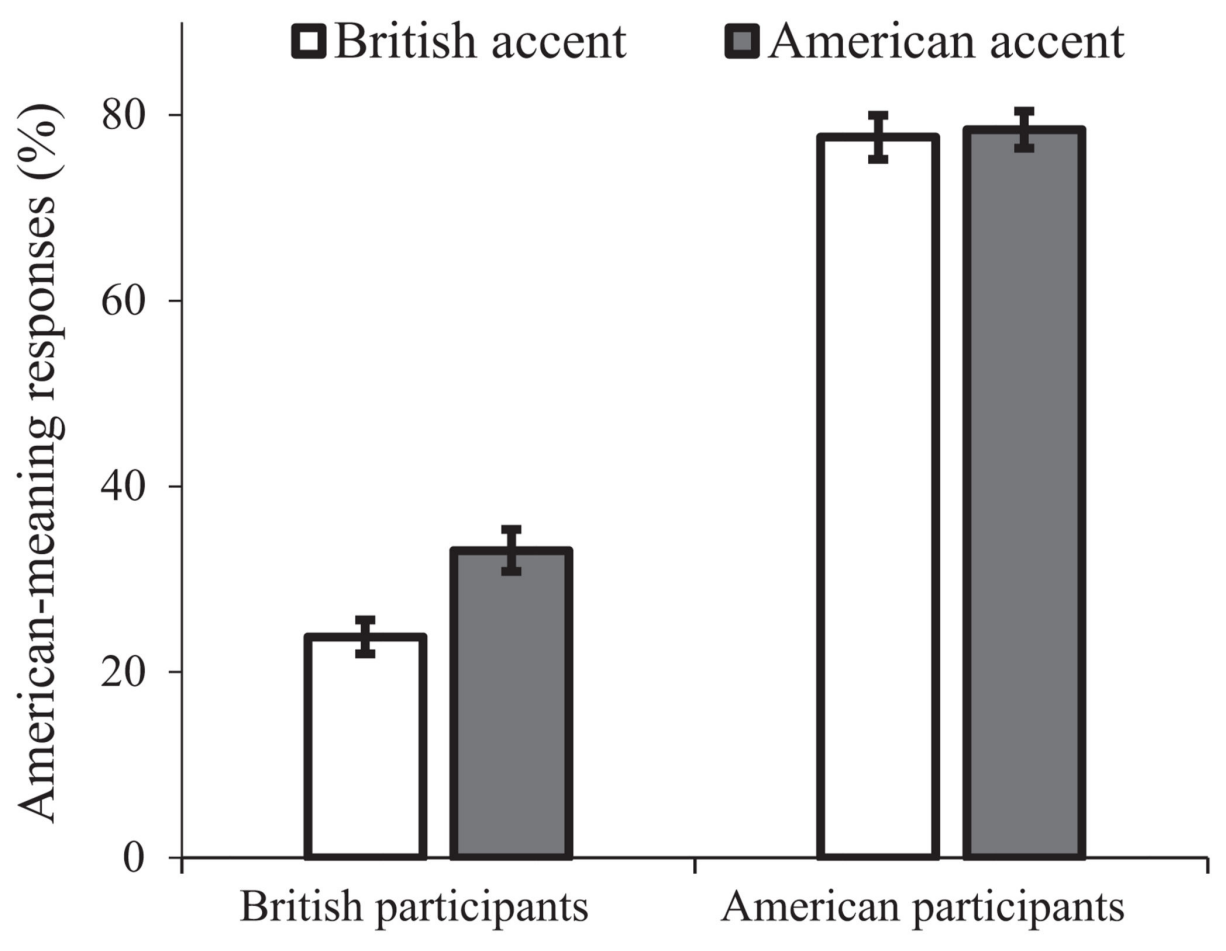
Proportion of American-meaning responses as a function of participant group and accent in Experiment 1. Error bars show ±1 SE based on participant means.

We next focused British- and American-meaning responses. Fig. 1 presents the percentage of American-meaning responses out of British- and American-meaning responses as a function of accent and participant group. We used logistic mixed effects (LME) modelling in our analyses and adopted the maximal random effect structure (in this and subsequent experiments) in order to guard against otherwise inflated likelihood of false positives (Barr, Levy, Scheepers, & Tily, 2013). All analyses were conducted using the lme4.0 package on R (version 3.0.2). We first examined how responses (British- vs. American-meaning responses) varied as a function of participant group (British vs. American participants) and accent (British vs. American accent). Predictors were contrast-coded (participant group: British participants = −0.5, American participants = 0.5; Accent: British accent = −0.5, American accent = 0.5). The LME model included participant group, accent, and their interaction as the fixed effects, and a maximal random effect structure by design (i.e. random participant and item intercept, plus random item slopes for group, accent, and their interaction).

The model revealed a significant main effect of participant group (*β* = 2.88, *SE* = 0.28, *z* = 10.34, *p* < 0.001): unsurprisingly, there were more American-meaning responses produced by American participants (78%) than by British participants (28%); such response pattern by the two groups reflected the fact that we used target words that have different dominant meanings for American and British English speakers. More importantly, accent also had a significant main effect (*β* = 0.33, *SE* = 0.14, *z* = 2.26, *p* = 0.024): in general, there were more American-meaning responses when participants listened to the American accent (58%) than to the British accent (48%). There was also a marginally significant interaction between group and accent (*β* = −0.51, *SE* = 0.27, *z* = −1.87, *p* = 0.062). We next conducted separate analyses for the two participant groups, using accent as the predictor. For British participants, accent produced a significant effect (*β* = 0.57, *SE* = 0.18, *z* = 3.14, *p* = 0.002), with more American-meaning responses when listening to American than British accent. Such a finding suggests that for British participants, meaning access for a word that has different dominant meanings between the British and American English is modulated by the accent the word is spoken in. For American participants, however, accent did not have a significant effect (*β* = 0.08, *SE* = 0.21, *z* = 0.38, *p* = 0.702). Thus, in contrast with British participants, American participants seemed to be relatively insensitive to the accent they heard when they accessed the meaning of an ambiguous word. Analysis of the self-reports of exposure to the non-native accent showed that British participants had high levels of experience with American English (2% of them reported to have encountered American English less than once a month, 5% once a month, 30% once a week and 63% once a day). In contrast, American participants were rarely exposed to British English (48% of them said to have encountered British English less than once a month, 11% once a month, 33% once a week, and only 8% once a day). The fact that American participants were rarely exposed to British English suggests that they might not have acquired the meaning dominances in British English for the target words we used here, which in turn explains the lack of an accent effect in this group.

We also explored the possibility that the accent effect might change over time. For instance, it is possible that the accent effect gradually accumulated as the experiment went by, resulting in a larger effect for later trials. To investigate this possibility, we conducted an exploratory analysis that included trial order (log-transformed) and its interaction with accent in the LME model reported above. If the accent effect changed over time we should expect an interaction between trial order and accent. The interaction between accent and trial order was nonsignificant for all participants, only British participants, and only American participants (|*z*|s < 1.2, *p*s > 0.10), suggesting that the accent effect did not significantly vary across the course of the experiment.

An additional exploratory analysis tested whether the accent effect would change as a function of the phonetic similarity between a word’s two accented instantiations. The accent effect of a word was calculated separately for the British and American participants as the difference in proportion of American-meaning responses between the two accents. Regression analyses showed that the accent effect did not significantly change as a function of a word’s phonetic similarity between accents as perceived by either British participants (*β* = 0.019, *SE* = 0.016, *t*(20) = 1.189, *p* = 0.248) or American participants (*β* = 0.026, *SE* = 0.021, *t*(20) = 1.21, *p* = 0.240). Thus there was no significant relationship between the strength of accent of a specific word and the word’s susceptibility to the accent effect on meaning access.

### 2.3 Discussion

We showed that listeners were more likely to retrieve the accent-congruent than accent-incongruent meaning; for instance, they were more likely to retrieve the American meaning of a word when the word was spoken in an American accent than in a British accent. This accent effect was significant overall, marginally larger for British than American participants and statistically unreliable for American listeners. This marginal interaction with nationality is not unexpected given American participants’ lower levels of experience with the British accent than British participants have with American-accented speech. Indeed, it has been shown that the processing of accented words is constrained by the experience a listener has with an accent (Sumner & Samuel, 2009): listeners with limited exposure to an accent are worse in recognising and representing words in that accent. Nonetheless, the effect of accent on word-meaning access was robust for British participants when considered in isolation. We anticipate that American listeners tested with more familiar accents (e.g. Northern and Southern US accents) or American listeners who have more exposure to British English would show effects of accent on word meaning access. We also note that the lack of an accent effect in the American participants does not conflict with the pre-test finding that American participants were more familiar with the American than British meaning for the target words: the non-significant accent effect simply suggests that our American participants were not sensitive to the different preferences by British and American speakers in using these meanings, at least when speech accent was the only cue to speaker identity.

The experiment thus provided the first demonstration that accent information is preserved beyond the level of identification of wordforms and influences word-meaning access. Such an accent effect is consistent with the speaker-model account that, parallel to lexical processing, listeners infer the dialectic background of the speaker from speech accent and use their knowledge of that dialect to boost accent-congruent meanings. The effect is also consistent with the surface-detail account in which the speech tokens themselves carry accent details that can feedforward via a lexical route and directly modulate word-meaning access. The surface-detail account, however, seems not to be supported by the observation that the magnitude of the accent effect did not vary as a function of the phonetic similarity of the two accented versions of a word. For instance, words such as “fall” and “quarter” were rated as sounding more dissimilar between the two accents than words such as “gas” and “mate”. However, the former words did not exhibit a significantly larger accent effect than the latter words. While we hesitate to draw strong conclusions from this null result, it might favour a speaker-model account. In addition, in light of the finding that the accent effect might be constant across the course of the experiment, it is likely that our (British) participants rapidly identified the dialectic identity of the speaker at the beginning of the experiment and consistently used such knowledge to interpret all words equivalently (see also Witteman, Weber, & McQueen, 2014).

To provide a stronger test of the speaker-model vs. surface-detail accounts, in Experiment 2 we directly manipulated the accent strength of target words using an audio morphing procedure (Rogers & Davis, 2017). In this way, we could create speech tokens that varied systematically in the strength of their accent (using morphs that were more or less strongly accented). A critical manipulation in this experiment was to use neutral accent morphs, i.e. tokens that could be heard as either British or American English depending on context. If the effect of accent on word-meaning access is driven by the acoustic characteristics of individual tokens, then strong-accent, but not neutral-accent tokens, should show changes in meaning preference. In contrast, if word-meaning access is modulated by an abstract representation of the dialectic identity of the speaker then neutral-accent tokens should show different meaning preferences provided they are interleaved with strong-accent tokens that inform listeners of the linguistic background of the speaker. Note that both the speaker-model account and the surface-detail account can be falsified by a positive rather than null finding in this experiment. On the one hand, if we observe that the neutral items differ depending on whether they are embedded within a block of British- or American-accented speech, this would constitute evidence against the surface-detail account, which predicts that these items should lead to similar responses between the two accent contexts. On the other hand, a significant difference between strong and neutral items within a single accent context would argue against the speaker-model account, which predicts that these two types of speech tokens should lead to a similar effect when perceived to come from the same speaker.

## 3 Experiment 2

### 3.1 Method

#### 3.1.1 Design

**Fig. 2:**
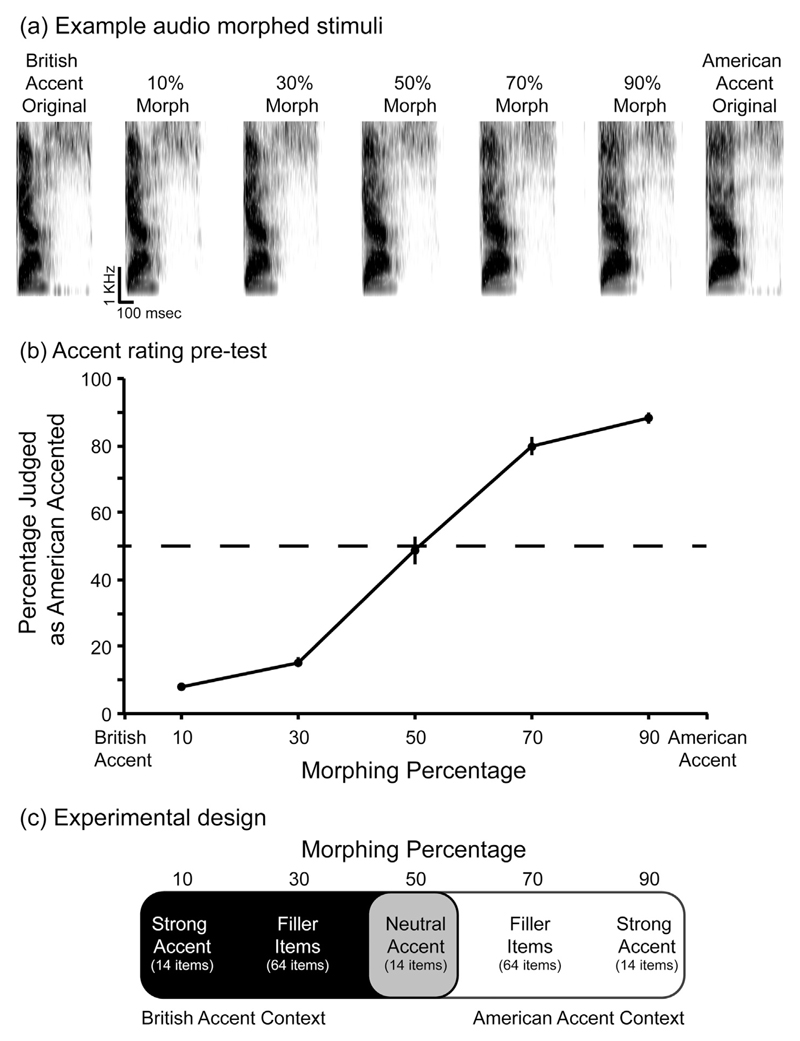
Stimuli, pilot data and method for Experiment 2. (A) Spectrograms of a single word “gas” spoken in a British Accent (leftmost panel), an American Accent (rightmost panel) and resynthesized at intermediate levels of accent strength using STRAIGHT software. (B) Results of the accent rating pre-test showing that 50% morph stimuli are perceived equally often as British or American (broken horizontal line shows 50% American responses). Error bars show ±1 SE over participants. (C) Design for Experiment 2 in which neutral-accent tokens are paired with strong-accent tokens in a British or American accent context.

We used STRAIGHT speech analysis and resynthesis software (Kawahara, Masuda-Katsuse, & De Cheveigne, 1999) combined with an audio-morphing procedure devised by Rogers and Davis (2017) to create acoustically-intermediate speech tokens between natural American and British accented recordings of isolated words (see Fig. 2A). This audio-morphing procedure is in many ways analogous to the well-established methods for morphing of photographic images (e.g. Calder, Young, Perrett, Etcoff, & Rowland, 1996), but applied to spoken words rather than faces. By morphing the British and the American versions of a word in varying proportions we created natural-sounding strongly-accented speech tokens (containing 90% of the acoustic properties of British accented and 10% of American accented speech tokens; or conversely 90% American and 10% British accented tokens) and neutral-accent speech tokens (morphed to contain 50% of the British and 50% of the American accented tokens).

Pilot testing (described below and summarised in Fig. 2B) suggested that, unlike the strongly-accented tokens, these neutral tokens were not consistently heard as having either a British or American accent. We created a British accent context or an American accent context for the neutral-accent morphs by intermixing them with either strong British accent morphs or strong American accent morphs, in a 2 (accent context: British accent vs. American accent; between participants) by 2 (accent strength: strong vs. neutral; within participants) design (Fig. 2C). Critically, in such a design, the neutral-accent morphs in the two accent contexts were exactly the same speech tokens, though they were intermixed with speech tokens of different accents. In addition, by manipulating accent context between participants and by using intermediate morphs of 70% British or American accent as fillers (to avoid the perception of a sudden accent shift; see below), we intended that all items would be perceived by the listeners as having been spoken by the same speaker. This was further reinforced by listening instructions and verified in a debriefing post-test described subsequently.

#### 3.1.2 Participants

As we observed an accent effect with British but not American participants in Experiment 1, we only tested British participants in this experiment (and indeed subsequent experiments). A total of 78 participants (paid £5) who had registered and reported themselves to be native speakers of British English were recruited from a UK-based crowdsourcing platform that provides participants for web-based experiments (Prolific Academic: http://www.prolific.ac). Three were excluded for correctly recognising fewer than 4 out of 8 English words in a vocabulary test (see below), one for failing to understand the word-association task (typing in the word heard rather than an associate), and one for living in the UK for only 8 years. Thus, data from 73 participants (average age = 27.9, SD = 9.8; 43 females; 35 participants in the British accent context and 38 in the American accent context) were analysed.

#### 3.1.3 Materials

The key materials were based on the word set used in Experiment 1. We excluded “football” (the two meanings are closely related) and “vest” (too many “other” responses). For the word “zip”, we replaced the rarely retrieved American meaning of “zero or nothing” with the American meaning of “postal code” (familiarity further tested; see below). We also removed some words whose British and American tokens generated unnatural-sounding intermediate morphed tokens. To make up for these exclusions, we carried out another pre-test on 28 additional potential stimuli using the same procedure (39 British, 26 American participants) and selection criteria as in Experiment 1 (see Appendix A for the ratings). One minor change from Experiment 1 was that we only required the two meanings of a word to each have an average rating greater than 2 in the familiarity ratings from British participants (but not necessarily from American participants); this was because in this experiment we would only test British participants. In terms of familiarity ratings for these words, there was a British meaning advantage for British English speakers (British meanings: *M* = 6.03, *SD* = 0.95, range = 2.54–6.82; American meanings: *M* = 4.89, *SD* = 1.06, range = 3.03–6.59). The stimuli were recorded in a further recording session with the same two female speakers used in Experiment 1. The resulting sound files were used to create morphs of different accent strengths, respectively 10%, 30%, 50%, 70% and 90% American accent (i.e. 90%, 70%, 50%, 30%, and 10% British accent). From these recordings, we selected 28 ambiguous target words (see Appendix A) and 71 unambiguous words (7 used as practice items and 64 as filler items).

Four experimental versions were created, two versions for the participants in the British accent context and two for participants in the American accent context, so that each word appeared once in each version (in either the strong- or neutral-accent condition, counter-balanced across two of the four versions). For the British accent context, each version contained 14 morphs with 90% British accent (strong-accent items), 14 morphs with 50% British accent (neutral-accent items), and 64 filler morphs with 70% British accent (see Fig. 2C). Similarly, for the American accent context, each version contained 14 morphs with 90% American accent (serving as strong-accent items), 14 morphs with a neutral accent (serving as neutral-accent items; these were the same speech tokens as in the neutral-accent condition in the British accent context), and 64 filler morphs with 70% American accent. In all versions, all items were presented in a random order with the constraint that two target items were always separated by 1–3 filler morphs; this was to disguise any detectable accent shift between the strong- and neutral-accent target items (e.g., from 50% British accent to 90% British accent) so that participants were more likely to perceive the spoken words as produced by the same speaker. Seven morphs with 70% British or American accent (depending on the accent context) served as practice items.

#### 3.1.4 Accent categorisation of morphs

To test whether the morphs were perceived as having the intended accent, 39 additional British participants were recruited from the same population as the main experiment, each being paid £4 after completing the online task (using Qualtrics). Each participant listened to morphs of varying accent strengths (i.e. 10%, 30%, 50%, 70% and 90% American accent) and made a two-alternative forced choice response to decide whether each token was pronounced in a British or American accent. Due to an experimenter error, one target word from the main experiment (“surgery”) was replaced with a different word (“pitcher”).

Participants largely categorized tokens as belonging to the British accent if they contained 10% or 30% American accent, and categorized as belonging to the American accent tokens containing 70% or 90% of the American accent (Fig. 2B). Neutral-accent morphs (50% mixtures of British and American accented tokens) elicited no significant bias towards either British or American accent responses (*β* = −0.06, *SE* = 0.22, *z* = −0.29, *p* = 0.774). As expected, participants did not consistently assign either a British or American accent to the neutral-accent morphs, making them liable to be heard as either accent depending on context.

#### 3.1.5 Procedure

The experiment was conducted online using Qualtrics. As in Experiment 1, participants first provided written responses to audio-morphed spoken words in a word association task, they then reported which meaning their response related to in a meaning clarification task, and finally completed a post-experiment questionnaire. Before the word association task, participants were told that they were about to listen to “Sarah” speak a series of words and that they would be performing a free association task based on these words before making judgements about “Sarah”. Together with the addition of the 70% morphs as fillers, this cover story was intended to prevent participants from noticing the accent strength manipulation and to increase the likelihood that they would perceive all the words (strong- and neutral-accent tokens) as coming from a single speaker. To assess this, after the meaning clarification task, we also asked participants to answer the question “How many speakers did you hear in the free association task?” by typing in a number.

### 3.2 Results

In responding to the post-experiment open question about the number of speakers they had heard in the word association task, 59 out of the 73 participants indicated that they had only heard one speaker, 12 participants indicated they had heard two speakers, 1 participant indicated they had heard three speakers, and 1 participant did not give an answer. This suggests that most of the participants believed our cover story that the spoken words were produced by a single speaker “Sarah”.

There were 1665 British- and American-meaning target responses and 379 “other” responses. An analysis comparing “other” responses with target responses revealed a main effect of accent, with more “other” responses in the American than British accent condition (52% vs. 48%; *β* = 0.71, *SE* = 0.26, *z* = 2.72, *p* = 0.006); critically, accent strength did not produce a significant main effect (*β* = −0.26, *SE* = 0.16, *z* = −1.66, *p* = 0.097) nor did it significantly interact with accent (*β* = 0.49, *SE* = 0.30, *z* = 1.63, *p* = 0.102) in the production of “other” responses.

**Fig. 3:**
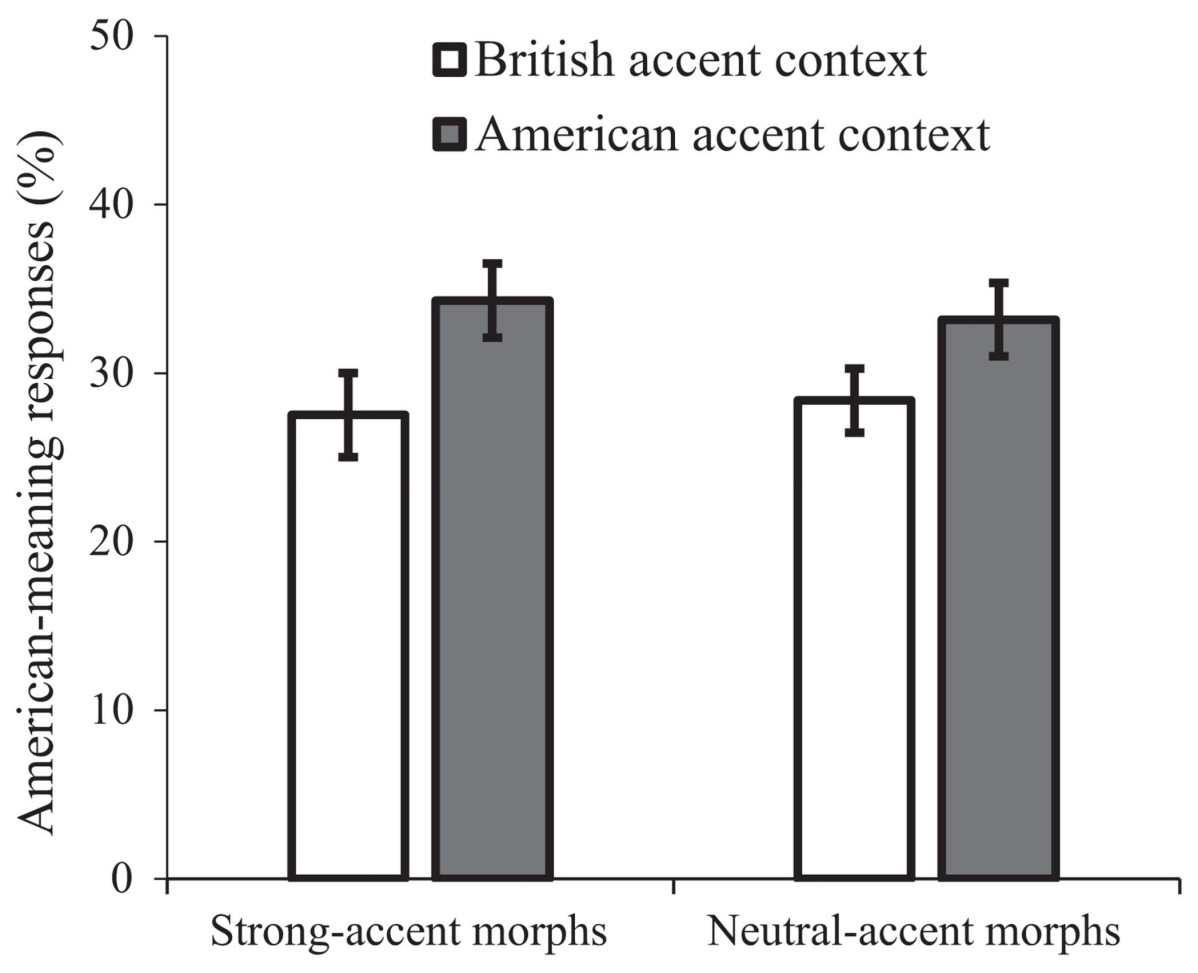
Proportion of American-meaning responses as a function of a morph’s accent strength and accent context. Error bars show ±1 SE based on participant means.

We plotted the mean proportion of American-meaning responses in each condition averaged over all participants in Fig. 3. We conducted an LME analysis on the responses, using accent context, accent strength, and their interaction as fixed effects. There was a main effect of accent context (*β* = 0.47, *SE* = 0.18, *z* = 2.57, *p* = 0.010), with participants tested in an American accent context producing more American-meaning responses (33%) than participants in the British accent context (28%). There was no significant main effect of accent strength (*β* = −0.07, *SE* = 0.15, *z* = −0.48, *p* = 0.632), and no significant interaction between accent context and accent strength (*β* = 0.12, *SE* = 0.28, *z* = 0.43, *p* = 0.670). For the two accent strengths, separate analyses showed that the strong-accent morphs in the American accent context (i.e. 90% American accent morphs) led to more American-meaning responses than the strong-accent morphs in the British accent context (i.e. morphs with 90% British accent) (*β* = 0.54, *SE* = 0.25, *z* = 2.17, *p* = 0.030), and critically, so did the neutral-accent morphs (*β* = 0.38, *SE* = 0.18, *z* = 2.10, *p* = 0.036), despite the fact that the same set of neutral-accent speech tokens (50% morphed stimuli) were used in both the British and American accent contexts. For the two accent contexts, separate analyses revealed that there was no difference in the proportion of American-meaning responses between strong- and neutral-accent morphs in the British accent context (*β* = −0.21, *SE* = 0.20, *z* = −1.01, *p* = 0.311) or in the American accent context (*β* = 0.01, *SE* = 0.18, *z* = 0.06, *p* = 0.956).

Subset analyses of the 59 participants who reported one speaker for all the speech tokens revealed a similar statistical pattern (significant main effect of accent context: *β* = 0.48, *SE* = 0.19, *z* = 2.51, *p* = 0.012; non-significant main effect of accent strength: *β* = −0.11, *SE* = 0.16, *z* = −0.68, *p* = 0.498; non-significant interaction: *β* = −0.08, *SE* = 0.30, *z* = −0.28, *p* = 0.780). Separate analyses revealed a marginally significant accent context effect for the strong-accent morphs (*β* = 0.46, *SE* = 0.24, *z* = 1.93, *p* = 0.054) and a fully significant effect for the neutral-accent morphs (*β* = 0.51, *SE* = 0.22, *z* = 2.35, *p* = 0.019). In addition, there was no difference between the two morph strengths either for the British accent context (*β* = −0.10, *SE* = 0.25, *z* = −0.39, *p* = 0.696) or the American accent context (*β* = −0.15, *SE* = 0.19, *z* = −0.78, *p* = 0.438).

We further explored the possibility that the accent effect changes over time by adding the main effect of trial order (log-transformed) and the interaction between accent context and trial order (as we did in Experiment 1). As in Experiment 1, the magnitude of the accent effect seemed to remain constant over the course of the experiment, as indicated by the non-significant interaction between accent context and trial order either when we included all the participants (*β* = −0.14, *SE* = 0.54, *z* = −0.26, *p* = 0.795) or when we included only those that reported hearing one speaker for the tokens (*β* = 0.35, *SE* = 0.49, *z* = 0.71, *p* = 0.480; the random slope of trial order for participants and the random slope for the accent context by trial order interaction for items were removed to achieve model convergence).

Finally, as in Experiment 1, we investigated whether the accent effect (for accented speech tokens) is affected by whether a particular word token contains strong acoustic cues to accent. Instead of using phonetic similarity ratings, however, we used accent discriminability scores. We calculated the percentage correct on this task when categorizing a British accented morph (i.e. morphs with 90% or 70% British accent) as belonging to the British accent and a American accented morph (i.e. morphs with 90% or 70% American accent) as belonging to the American accent (Neutral-accent morphs were excluded because there was no “correct” categorisation for these morphs). A word is classified as more discriminable if its morphs were categorized more accurately across the four accent strengths (see Appendix A). Thus the accent discriminability scores reflected how accurately participants could categorise the accented form of each word. We thereby tested whether the accent effect on word meaning access is stronger for those words for which it is easier to discriminate between the two accents (e.g., “bonnet” and “jumper” as compared to “mate” and “dummy”). Despite using a more direct behavioural measure of accent discriminability (rather than phonetic similarity as in Experiment 1), regressing a word’s accent effect on its discriminability score still did not yield a significant relationship (*β* = −0.20, *SE* = 0.55, *t*(26) = −0.37, *p* = 0.717). Thus, contra to the prediction of the surface-detail account, and consistent with the accent effect for neutral accent tokens, there was again no effect of the accent strength of individual words on the size of the word-meaning effect.

### 3.3 Discussion

The results replicated the finding in Experiment 1 that hearing a word in a particular accent facilitates access to the accent-congruent meaning. Most importantly, two findings provide direct support for the view that listeners use an accent context as part of a speaker model that guides meaning access and are inconsistent with an account in which the surface details of specific words drive changes in meaning access. First, neutral-accent speech tokens created with audio-morphing were more likely to be interpreted with the American dominant meaning if they were mixed with American accented trials, than if they were mixed with British accented trials. Thus, the same set of speech tokens was interpreted by listeners in different ways depending on the accent characteristics of other tokens within the same experimental list. This finding constitutes *positive* evidence against the surface-detail account of the accent effect, which predicts the neutral-accent items should lead to similar responses between the two accent contexts. Indeed, there was no significant difference in the strength of the accent effect between strong and neutral accented trials within the same accent context. A second finding from this experiment (as in Experiment 1) is that there was no significant correlation between the salience of accent cues in individual words and the size of the accent effect for these words.

These two findings instead provide evidence in favour of a speaker-model account in which listeners determine the accent of the person speaking (perhaps early on in the experiment) and use this speaker knowledge as an additional and consistent context to bias the interpretation of subsequent words in the experiment. Note that in this experiment, unlike in Experiment 1, evidence for this speaker-model context (and against surface-detail mechanism) was based on a positive rather than a null finding. It is striking that the same neutral-accent speech tokens led to significant shifts in interpretation to the same degree as strong-accent speech tokens, solely by virtue of being heard among accented speech tokens in the same experimental version. These findings support the conclusion that accent information modulates meaning access by creating a speaker-level accent context against which subsequent words are interpreted. The finding that the accent effect did not significantly increase in magnitude across the experiment also suggests that the speaker’s accent was identified very early in the experiment and that this knowledge influenced meaning retrieval equivalently for all remaining trials in the experiment. Future experiments are needed to determine exactly how rapidly this effect can emerge.

Although these results fit with a speaker-model account of accent effects, there is still an alternative way in which they could be explained. Listeners are clearly extracting some sort of accent representation across different tokens in the experiment, such that this knowledge can bias the interpretation of accented and neutral-accent words belonging to the same speaker equally. But it is also possible that the mechanism underlying this bias is not speaker-specific, or even speech-specific. Instead, it could be that a more abstract concept of one or other geographical location is being activated in the mind of the listener (i.e. a concept of Britain or America as places rather than individual speakers coming from either country) and might reflect a bias that has a more pervasive effect on the interpretation of all incoming linguistic material. This kind of more generalised geo-cultural bias has previously been found in the perception of synthesized speech, in which context was manipulated by the presence of different stuffed toys in the experimental room (Hay & Drager, 2010). Participants from New Zealand who listened to speech in the context of stuffed kangaroos and koalas (associated with Australia) tended to interpret the speech as more Australian-like, whereas in the context of stuffed kiwis (associated with New Zealand) listeners tended to judge synthetic speech as being New Zealand-like.

In Experiment 3, therefore, we tested the generality of accent context effects by observing whether they extended to written words that were interleaved with accented spoken words. Unlike neutral-accent morphs, written words cannot be assimilated to “sound” like strongly accented spoken words and hence are likely to be treated distinctly from accented spoken words (i.e. not coming from the same speaker). If the accent context is part of a speaker model that listeners establish for the person speaking, then we should expect that this accent context would apply to spoken words that listeners hear as coming from the same speaker, but not for written words in the same experimental block. However, if the accent context effect derives from a more general geo-cultural bias towards American or British word meanings then the word meanings retrieved should be affected in an equivalent way for written and spoken presentations. For instance, if the American accent in the spoken words instigates a sense of Americanism such that listeners are biased towards the American meaning during word recognition, then we should expect the geo-cultural bias to be applied to all words in the same experimental context, whether spoken or written. We also included an additional, written-only condition in the experiment to obtain a baseline measure of the word meanings that listeners retrieve in the absence of hearing any spoken words.

## 4 Experiment 3

### 4.1 Method

#### 4.1.1 Design

We created two between-participant accent contexts: a British accent context in which half of the words were spoken in a British accent and the other half written, and an American accent context in which half of the words were spoken in an American accent and the other half written. In addition, we included a written-only baseline context, where all the words were presented visually. Free from any the influence of the accent in spoken words, this condition thus enabled us to assess the magnitude of the accent effects for the spoken and written words (if any) in the two accent conditions. The three contexts (British accent, American accent and written-only) were manipulated between participants while modality (spoken vs. written words) was manipulated within participants for the British and American accent contexts. The British and American accent contexts each had two versions (between which the target words were counter-balanced between the two modalities) while the written-only context had only one version.

#### 4.1.2 Participants

Native speakers of British English were recruited from Prolific Academic and UCL Year 1 undergraduate psychology students. After exclusion based on the criteria in Experiment 1, there were 153 participants (average age = 28.3, SD = 14.9; 99 females), with 65 randomly assigned to the two versions of the British accent context, 54 to the two versions of the American accent context, and 34 to the written-only context. Participants from Prolific Academic participated for a reward of £4 and UCL psychology students participated as a course requirement.

#### 4.1.3 Materials

Materials from Experiment 1 were used except for the three words (“football”, “vest”, “zip”) which were excluded in Experiment 2 for reasons related to participants responses; we further removed two words with culture-specific American meanings that British participants rarely retrieved (i.e. “quarter” and “nickel”). We added three words that were newly introduced in Experiment 2 (“bonnet”, “dummy”, and “nicked”). Therefore, we had 20 ambiguous target words (see Appendix A). In terms of familiarity ratings for these selected words, there was a British meaning advantage for British English speakers (British meanings: *M* = 6.23, *SD* = 0.62, range = 4.91–6.81; American meanings: *M* = 4.65, *SD* = 0.96, range = 3.28–6.38). We also used 20 of the filler words from Experiment 1.

#### 4.1.4 Procedure

This was the same as in Experiment 1 except that half of the words in the British and American accent contexts and all the words in the written-only context were presented visually on the screen rather than in spoken form.

### 4.2 Results

There were 2591 British- and American-meaning target responses and 469 “other” responses across all the conditions. An analysis comparing “other” responses with target responses revealed a main effect of modality, with more “other” responses for the spoken than written words (18% vs. 12%; *β* = −0.92, *SE* = 0.18, *z* = −5.19, *p* < 0.001), but no significant effects of accent (*β* = 0.24, *SE* = 0.19, *z* = 1.30, *p* = 0.192) or interaction (*β* = −0.14, *SE* = 0.30, *z* = −0.46, *p* = 0.649).

**Fig. 4:**
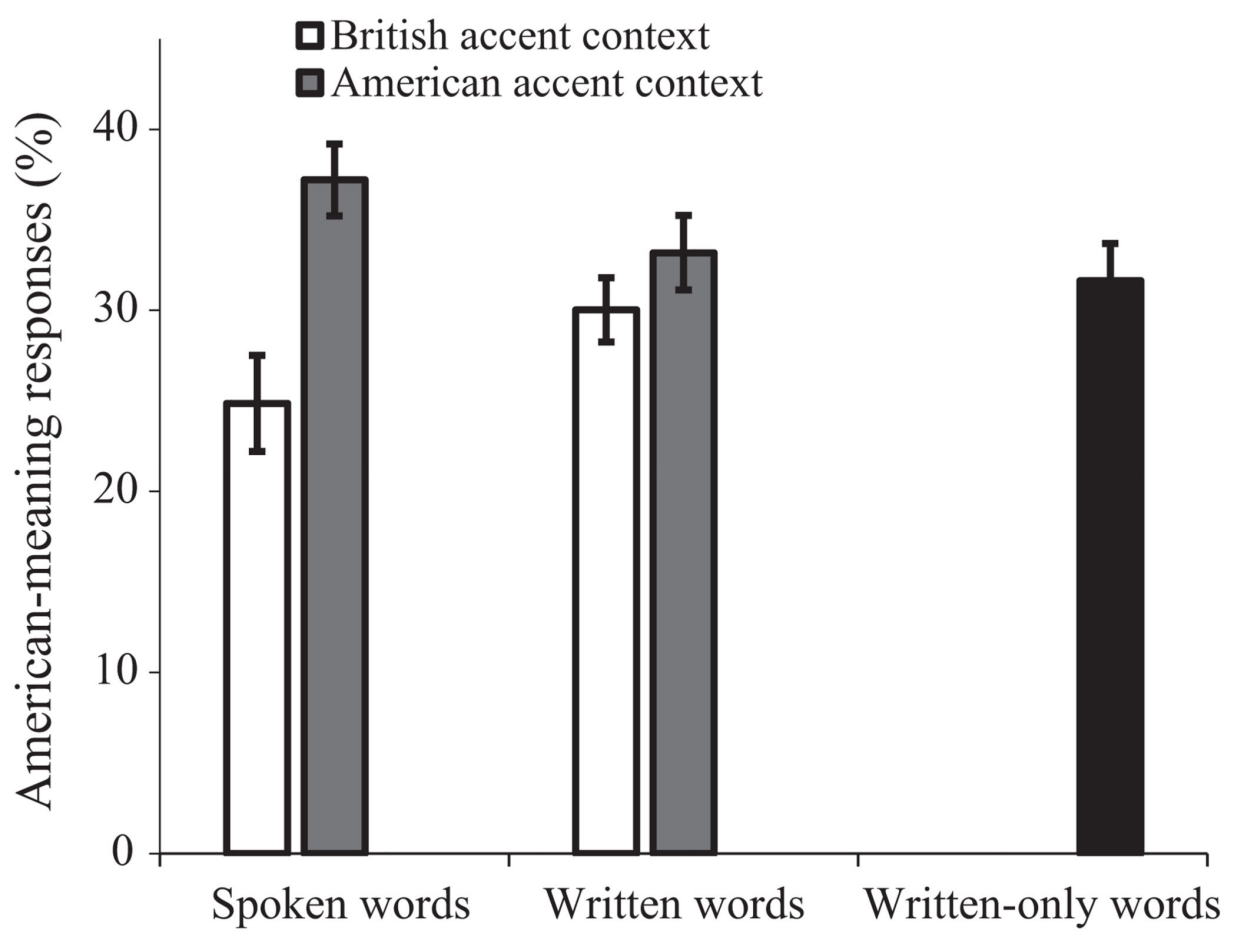
Proportion of American-meaning responses as a function of word modality and accent context. Error bars show ±1 SE based on participant means.

Fig. 4 presents the mean proportion of American-meaning responses from each of the conditions. To examine whether the accent effect (if any) is modulated by modality (spoken vs. written), we first conducted a 2 (accent context: British accent vs. American accent) * 2 (modality: spoken vs. written) LME analysis on data from the British and American accent contexts, (i.e. excluding the written-only baseline context). There was a significant main effect of accent context (*β* = 0.45, *SE* = 0.14, *z* = 3.24, *p* = 0.001), indicating that there were more American-meaning responses in the American accent context than in the British accent context (35% vs. 28%). Modality did not produce a significant main effect (*β* = 0.04, *SE* = 0.12, *z* = 0.36, *p* = 0.718), but there was a significant interaction between accent context and modality (*β* = −0.65, *SE* = 0.25, *z* = −2.58, *p* = 0.010), showing that the accent effect was significantly larger for spoken words than for printed words. Separate analyses on the two modalities showed that, for spoken words, accent context had a significant effect (*β* = 0.69, *SE* = 0.16, *z* = 4.29, *p* < 0.001): Participants produced more American-meaning responses when listening to words spoken in the American accent than in the British accent. However, though written words induced numerically more American-meaning responses in the American than British accent context (33% vs 30%), this difference was not statistically significant (*β* = 0.21, *SE* = 0.15, *z* = 1.35, *p* = 0.177); that is, the accent effect observed for spoken words did not transfer to written words presented during the same test session.

It is important to note that, in the null hypothesis significance tests we used, the absence of evidence for an accent effect in the written words does not necessarily constitute evidence of the absence of such an effect. To further explore the null effect, we turned to Bayes factor analysis, which enabled us to, given the observed data, quantify the likelihood of the null hypothesis against that of the alternative hypothesis (BF_01_) or the likelihood of the alternative against that of the null (BF_10_) (Jeffreys, 1998; Kass & Raftery, 1995; Wagenmakers, 2007). Following Wagenmakers (2007), we used the difference in Bayesian Information Criterion (BIC) between two competing hypotheses (LME models) to compute the Bayes factor (i.e. BF = e^DBIC/2^). For the written words, the Bayes factor analysis showed that, given the data, the null hypothesis (i.e. the accent context does not affect meaning access for the written words) was 12 times more likely than the corresponding alternative hypothesis (i.e. the accent context applies to meaning access for the written words; BF_01_ = 12.2). In contrast, for the spoken words, the alternative hypothesis (i.e. the accent context impacts word meaning access for the spoken words) was 245 times more likely than the corresponding null hypothesis (i.e. the accent context does not impact word meaning access for the spoken words; BF_10_ = 244.7), given the data. The Bayes factor analyses thus confirmed our hypotheses that while accent context modulates meaning access for spoken words, it does not modulate meaning access for written words that were intermixed with the spoken words.

Next, we compared responses to spoken and written words in the two accent contexts with words in the written-only context (which served as a baseline) to further test for an accent context effect on responses to written words. Note, that the baseline written-only context contained no explicit cues to indicate whether American or British meanings were required, but since this condition was tested in the UK on British participants we would have expected primarily British responses. Predictors were treatment-coded. Compared to written words presented in the written-only context, spoken words in the American accent context led to significantly more American-meaning responses (*β* = 0.35, *SE* = 0.16, *z* = 2.21, *p* = 0.027), and spoken words in the British accent context led to significantly fewer American-meaning responses (*β* = −0.40, *SE* = 0.20, *z* = −1.97, *p* = 0.049). These findings suggest that both of the British and American accents played a role in guiding meaning access – i.e. the British accent reduced and the American accent increased the availability of the American meanings for retrieval. Written words, on the other hand, elicited statistically indistinguishable numbers of American-meaning responses between the British accent and written-only contexts (*β* = −0.02, *SE* = 0.17, *z* = −0.11, *p* = 0.912) and between the American accent and written-only contexts (*β* = 0.06, *SE* = 0.19, *z* = 0.32, *p* = 0.748). These results further confirm that the accent context created by spoken words does not generalise to written words presented in the same experimental setting as the spoken words.

As we only observed an accent effect with spoken words, we next conducted a trial order analysis on the spoken items only in order to investigate whether the magnitude of the accent effect varied as a function of trial order by including the accent context, trial order (log-transformed) and their interaction in the LME model. As in Experiments 1 and 2, the interaction did not reach significance (*β* = 0.80, *SE* = 0.59, *z* = 1.37, *p* = 0.172), suggesting that the magnitude of the accent effect for the spoken words remained constant across the experiment. While this lack of a modulatory effect of trial order on the accent effect is consistent with trial order findings in the previous experiments, the null effect in individual experiment could be due to insufficient power. To resolve this issue, we combined all the data for which an accent effect was observed (i.e. data from British speakers in Experiment 1, all the data from Experiment 2, and data for the spoken words in Experiment 3). For the sake of simplicity, we only included the fixed effect of accent (American vs. British accent), the fixed effect of trial order (log-transformed) and their interaction in the LME modelling of British- vs. American-meaning responses. As expected, accent had a highly significant effect, with more American-meaning responses when a word was spoken in an American than British accent (or accent context) (34% vs. 26%, *β* = 0.60, *SE* = 0.12, *z* = 5.08, *p* < 0.001); trial order did not produce a significant effect (*β* = 0.004, *SE* = 0.11, *z* = 0.03, *p* = 0.974) and did not modulate the accent effect (*β* = −0.06, *SE* = 0.22, *z* = −0.29, *p* = 0.774). This latter finding is consistent with the findings from previous individual analyses that the accent effect did not vary in magnitude across the course of the experiments.

### 4.3 Discussion

Words were more likely to be interpreted with their American meanings if they were spoken in an American than in a British accent, replicating the accent effect observed in Experiments 1 and 2. However, the accent effect observed in spoken words did not generalise to written words (as they did to neutral-accent spoken words in Experiment 2) interleaved into the same experimental version. Listeners were no more likely to access the American meanings for written words when they were intermixed with American-accented spoken words than when mixed with British-accented words. Furthermore, written words elicited similar proportions of American-meaning responses regardless if they occurred in an (British or American) accent context or in a written-only context: while listeners appear to establish an accent context that they used to interpret spoken words, they do not extend that accent context to interleaved written words.

We take this pattern of results to argue against the more generic, geo-cultural bias account that we discussed previously, whereby the activation of ‘American-ness’ or ‘Britishness’ has a pervasive impact on all incoming linguistic material. This generic bias can certainly explain the results of Experiments 1 and 2, and the present finding that hearing British-accented spoken words increased the number of British meanings generated in the same way that hearing American-accented words increased the number of American meanings. However, according to this more general bias account, an abstract accent context should equally influence meaning access for written words. The lack of an accent effect that extends to the written words suggests that this generic bias account is not correct.

Instead, the results are consistent with a speaker-model account that the influence of accent on word meaning access is tied to an underlying model of the person speaking. That is, listeners use their dialectic background inference about a speaker to interpret words that are believed to be produced by the speaker in question. Unlike the neutral-accent morphs in Experiment 2, written words cannot be assimilated to “sound” like the (British or American) accented words; in addition, there is no reason for listeners to believe that the written words and the spoken words came from the same source (i.e. the same speaker). Therefore, the speaker model is not applied to the interpretation of written words, hence the lack of an accent effect. Finally, we also replicated the finding that the magnitude of the accent effect on the spoken words did not vary during experiment, suggesting that the speaker model was established very early on during the experiment.

## 5 Experiment 4

Using the word association paradigm, three experiments reported above showed that listeners use accent information to establish the dialectic identity of the speaker, against which they interpret words coming from that speaker. However, the off-line nature of word association (i.e. participants can take as much time as they like to contemplate their response) means it is hard to draw any conclusions regarding the time-course of accent modulation in meaning access. It is possible that accent is used on-line to modulate activation and selection of the alternative meanings of an ambiguous word such that accent-congruent meanings are boosted in their activation, leading to a higher chance of being selected; alternatively, it is also possible that the speaker model is used in off-line, post meaning access. In the latter case, an ambiguous word such “bonnet” would activate both of its meanings (e.g., Duffy et al., 1988; Swinney, 1979) but this activation and the subsequent meaning selection would not differ as a function of the accent the word is spoken in. With both meanings available, the listener could then use accent information (and the speaker model) to select an interpretation.

While the finding of accent modulation of meaning access, strategic or not, is in itself interesting, it is important to find out whether accent is used early on to modulate meaning access in on-line language processing. The strategic use of accent post meaning access would require off-line contemplation which consumes time and cognitive resources (e.g., thinking about which meaning is more appropriate given the accent and other speaker-specific information). Thus, though people can afford the time and resources to strategically contemplate about accent-meaning congruency in off-line tasks such as word association, this strategy is unlikely to influence on-line language processing tasks that require rapid meaning selection. If, instead, accent is used on-line to modulate meaning activation and selection, we should expect accent to facilitate the retrieval of accent-congruent meanings even in speeded tasks.

In this experiment, we conducted a speeded semantic relatedness task in which participants first heard a target word (e.g., “bonnet”) spoken in either the British or American accent and then at the immediate offset of the word saw a probe word that related to either the British or American meaning (e.g., “CAR” vs “HAT”). Their task was to decide, as quickly and accurately as possible, whether or not the visually presented probe word was semantically related to the accented word they had heard. If an accent effect is evident in speeded responses made soon after the offset of the accented words, then this would suggest on-line modulation of meaning access based on speaker accent. We predict that British meanings should be responded to more quickly and accurately compared with the American meanings, which are less frequent (i.e. subordinate) for our British English speaking participants. For instance, responses to the British-meaning probe “CAR” should be faster and/or more accurate than to the American-meaning probe “HAT” following the target word “bonnet”. More importantly, if speaker accent modulates on-line meaning access, we should expect quicker and more accurate responses when there is a congruency between the speaker accent and the probed meaning (a British/American-accented target word with its British/American meaning probed) than when there is not (a British/American-accented target word with its American/British meaning probed). In other words, we should expect an interaction between speaker accent and probed meaning as a result of accent modulation of on-line meaning access.

As quite a few studies have recently demonstrated the feasibility of precise stimuli presentation timing and high-quality reaction time data collection when testing participants remotely using web-based experimental platforms (Hilbig, 2016; Reimers & Stewart, 2015, 2016), the current experiment was conducted online, using Qualtrics survey software and a JavaScript reaction-time engine (Barnhoorn, Haasnoot, Bocanegra, & van Steenbergen, 2015).

### 5.1 Method

#### 5.1.1 Design

As in Experiment 1, we manipulated speaker accent (British or American) between participants (i.e. a participant was exposed to only one accent) but within items; additionally we counterbalanced, within participants and items, the probed meaning (British or American) of the ambiguous word. The set of experimental items was split into two lists in order to balance the assignment of items to probed meaning conditions. The combination of the accent manipulation and probe counterbalancing produced four versions of the experiment.

#### 5.1.2 Participants

A total of 133 British English-speaking participants were recruited from Prolific Academic, using the same eligibility criteria as in Experiment 1. They all had an IP address located in the UK, and an adequate internet connection speed. 11 of the participants experienced technical problems during the experiment which prevented them from finishing, and 2 reported having lived in the USA for more than 3 months, leaving 120 participants (average age = 29.8, SD = 8.2; 69 women; 30 participants per version). All participants were compensated £1.50 for their time.

#### 5.1.3 Materials

Each item consisted of a spoken target word followed by a written probe word (in upper case), either related or unrelated to a meaning of the target. 20 experimental targets (see Appendix A) were selected from the set of pre-tested ambiguous words used in Experiments 1–2. For a target word to be included, its two meanings had to be sufficiently different as to allow the selection of probe words that clearly relate to one meaning but not the other (e.g. “chips” was excluded because both meanings are types of food). For the selected words, the British meaning was rated as more familiar by British English speaking participants: (British meaning: *M* = 6.09, *SD* = 0.75, range = 4.64–6.81; American meaning: *M* = 4.92, *SD* = 1.04, range = 3.28–6.38).

Two probe words were selected for each experimental target, one relating to the British meaning and one to the American meaning. Probe words were selected from word association responses from previous experiments, and from the Edinburgh Associative Thesaurus (Kiss, Armstrong, & Milroy, 1972). It was not necessary to closely match the two probe words for each item because we were interested in the interaction between accent and meaning rather than the main effect of probed meaning. Nonetheless there were no significant differences in probe word length in terms of number of letters (mean difference = 0.0, SD = 2.3, range = −4 to 5; *t*(19) = 0.00, *p* > 0.999), log frequency per million (mean difference = 0.3, SD = 0.9, range = −1.2 to 2.3; *t*(19) = 1.41, *p* = 0.176) or context diversity (measured as the number of films/televisions a word appeared in; mean difference = 0.3, SD = 0.8, range = −0.9 to 2.1; *t*(19) = 1.53, *p* = 0.143) between the British- and American-meaning probes (log frequency and context diversity obtained from the SUBTLEX-UK database; van Heuven, Mandera, Keuleers, & Brysbaert, 2014).

48 relatively unambiguous filler targets were included to balance the number of related and unrelated responses and to reduce the salience of meaning ambiguity: 34 were paired with an unrelated probe (e.g. snake-CLOUD) and 14 were paired with a related probe (e.g. town-CITY). As the experimental materials were selected from tokens that were recorded in different sessions (albeit with the same speakers in the same recording booth), all sound files were matched for root-mean-squared amplitude to prevent differences in sound quality. All silences before or after words in the sound files were identified and removed manually to allow for the precise timing of spoken word onset and, more importantly, to ensure that the probes were presented immediately upon the offset of the target words.

#### 5.1.4 Semantic relatedness pre-test

We pre-tested the relatedness of all the target-probe pairs (experimental and filler) in order to ensure that the associations were sufficiently clear, given one specific meaning of the target. Two versions were produced by crossing the assignment of experimental target and (British- or American-meaning) probes so that participants saw each target only once. 22 participants (11 per version, none of whom took part in the main experiment) saw the target along with a short definition (either the American or British meaning for experimental targets) and the probe word, e.g., FALL (to trip over something) – INJURY. We instructed participants to only consider the defined meaning of the first word when rating the strength of the relationship between the two words on a 7-point scale ranging from 1 (completely unrelated) to 7 (strongly related). A second pre-test (N = 11) was run to test alternatives for poor-performing probes (i.e. low ratings for related pairs, high ratings for unrelated pairs), and for these items the probes with the most extreme related/unrelated ratings were selected. The ratings for the final set of word pairs were highly polarized, with related pairs rated well above the mid-point of the scale (experimental items: *M* = 6.3, *SD* = 0.5, range = 5.3–7.0; related fillers: *M* = 6.4, *SD* = 0.3, range = 5.6–6.9) and unrelated filler pairs well below (*M* = 1.1, *SD* = 0.2, range = 1.0–1.8).

#### 5.5.5 Procedure

The experiment was run using Qualtrics and the Qualtrics Reaction Time Engine (QRTE; Barnhoorn et al., 2015). Participants were told that they would hear a word and then immediately see a word on the screen, and their task was to indicate as quickly and accurately as possible whether or not the meanings of the two words were related. They were then given examples of semantically-related and unrelated word pairs along with short explanations for each.

The experiment began with a practice block followed by the main experiment. Each trial consisted of a 500 ms fixation cross followed by the presentation of the spoken target word (with the fixation cross still on the screen). Immediately upon the offset of the spoken target, the written probe word replaced the fixation cross in the centre of the screen, along with the response cues beneath (“Unrelated” and “f” on the left-hand side of the screen, “Related” and “j” on the righthand side). This display remained on the screen until a valid response was detected. If the response latency was longer than 2 s then a screen was shown indicating that the response was too slow, and reminding participants to respond as quickly and as accurately as possible. The inter-trial interval was 1 s. Though we used a connection speed check at the start of the experiment to minimise variation of the interval, we noted that the interval might occasionally increase slightly due to intermittent connection problems. However, the timing of stimulus presentation within each trial was never affected by connection speed problems, as all stimuli were loaded from the server before the start of each trial. The main experiment always began with 6 filler trials (half related, half unrelated), followed by the remaining 62 trials presented in random order.

### 5.2 Results

**Fig. 5:**
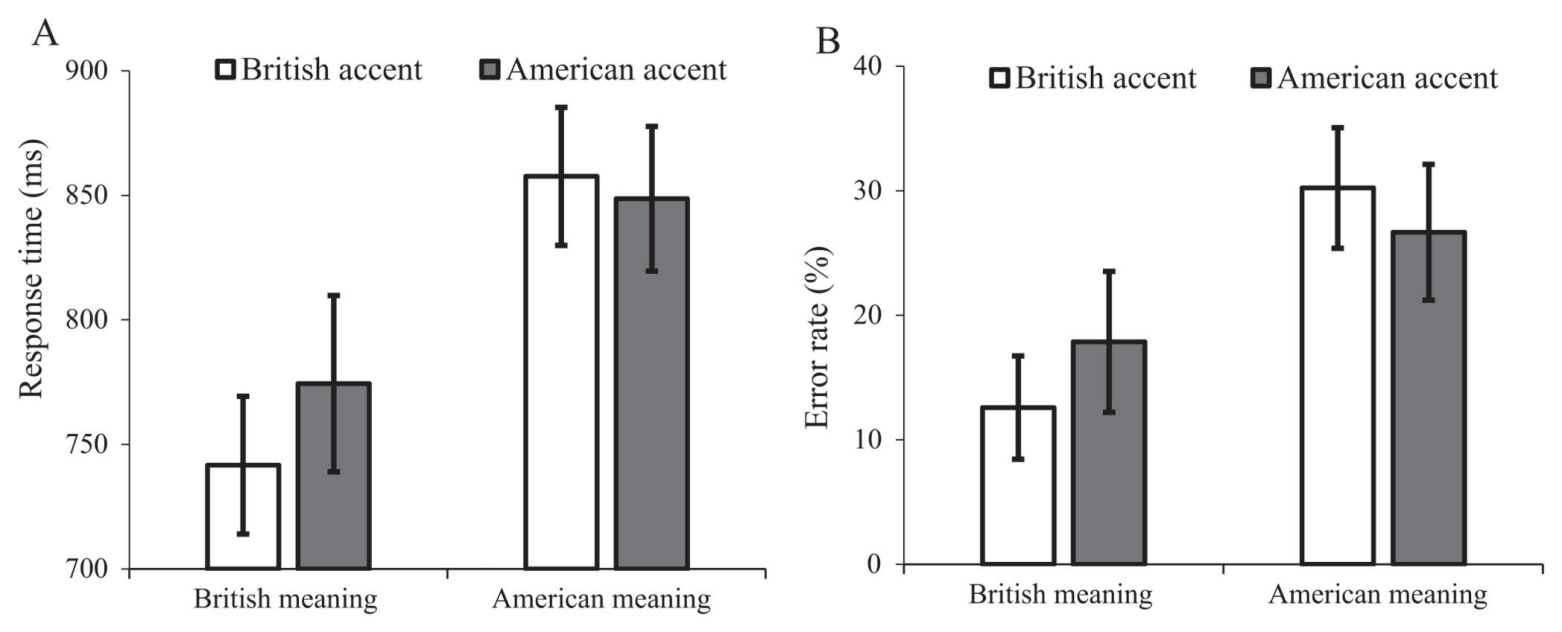
Response times (A) and error rates (B) in Experiment 4. Error bars show ±1 SE, adjusted to show within-participant effects of probe meaning (Morey, 2008).

Of the 2400 experimental trials (120 participants × 20 items), any trials with RTs less than 300 ms or greater than 2500 ms (6 trials; 0.3%) were excluded on the assumption that very fast responses reflect accidental key presses and very slow responses reflect lapses in attention. For the RT analysis, all incorrect responses were also removed, leaving 1871 trials (78.2%). Using an inverse-transform of the raw RTs (-1000/RT), the model met the assumptions of normally-distributed residuals and homogeneity of variance, so no further data trimming was needed (Baayen & Milin, 2010). Mean response times as a function of condition are shown in Fig. 5A. As in previous experiments, predictors were contrast-coded for linear mixed effects analyses (Accent: American = 0.5, British = −0.5; Meaning: American = 0.5, British = −0.5). As linear mixed-effects models (unlike logistic mixed-effects models) do not return *p*-values, we used likelihood ratio tests to determine the significance of a fixed effect, using backward model comparisons (i.e. by comparing the full model with a model without a fixed effect, everything else being the same).

The results showed that accent did not produce a significant effect (*χ*^2^(1) = 0.23, *p* = 0.629): response times were similar regardless of the accent a target word was spoken in. There was, however, a significant main effect of meaning (*χ*^2^(1) = 6.26, *p* = 0.012) such that (as expected) response times were faster if a target was followed by a probe relating to the British meaning compared to American meaning (a British meaning advantage). Crucially, the interaction between accent and meaning was also significant (*χ*^2^(1) = 4.08, *p* = 0.044). This interaction revealed effects of accent-meaning congruency: as shown in Fig. 5A, responses were faster when the probed meaning was congruent with the accent than when it was not. This finding therefore suggests that speaker accent facilitates on-line access to accent-congruent meanings.^2^

The error analysis included 2394 trials (i.e. excluding 6 trials with RTs < 300 ms or >2500 ms). Percentages of errors by accent and meaning are shown in Fig. 5B. LME analyses showed a similar statistical pattern for the error rate data as for the response time data. Accent did not produce a significant effect (*β* = −0.27, *SE* = 0.17, *z* = −1.59, *p* = 0.111), but the effect of meaning was significant (*β* = −1.08, *SE* = 0.33, *z* = −3.23, *p* = 0.001), with fewer errors when a target was followed by a British- than American-meaning probe. Critically, the interaction between accent and meaning was significant (*β* = 0.77, *SE* = 0.27, *z* = 2.83, *p* = 0.005). Again, as shown in Fig. 5B, this interaction showed accent-meaning congruency effects: there were fewer errors in conditions where the accent and the probed meaning matched than in conditions where they did not match.^3^

### 5.3 Discussion

Experiment 4 showed that for a speeded task in which participants had to decide whether a probe was semantically related to a target word, there was an advantage of accent-meaning congruency: responses to a particular meaning were quicker and more accurate if that meaning was congruent with the speaker accent. In other words, congruency between accent and meaning enhanced the speed and accuracy of meaning access. These results thus replicate the finding in Experiment 1 that accent modulates meaning access; importantly this was shown in a task that tapped into rapid semantic processing which is uncontaminated by subsequent contemplation of alternative meanings. The results therefore suggest that the accent in which a word is spoken influences the on-line retrieval of word meanings. This conclusion is consistent with the recent demonstration that accent information is used on-line for monitoring whether a lexical choice is appropriate in a dialect (Martin et al., 2016).

## 6 Experiment 5

We have shown in Experiment 4 that accent information is used on-line to facilitate the retrieval of accent-congruent meanings. However, since that experiment and indeed Experiments 1–3 used meta-linguistic tasks that test single-word comprehension, one may also ask whether accent modulation on meaning access extends to more ecological language comprehension when ambiguous words are used in sentences. For instance, to understand the sentence “The mechanic needed to repaint the whole bonnet”, one will need to retrieve its British meaning (car part) instead of its American meaning (hat). If accent modulates meaning access in sentence comprehension, we should expect quicker and more accurate interpretation of sentences in which accent and meaning are congruent.

Experiment 5 thus used a sentence interpretation task, during which participants listened to a sentence and decided whether it made sense or not. The sentences were spoken in either a British or American accent and, in the target trials, the final word of the sentence was an ambiguous word that was disambiguated towards either the British meaning (e.g., “The mechanic needed to repaint the whole bonnet”) or the American meaning (e.g., “The woman decided to iron her daughter’s bonnet”). As in Experiment 4, we expected (British) participants to be faster and more accurate when the British meaning was used. More critically, if listeners make swift use of accent information (via a speaker model) at the level of sentence comprehension, then we would also expect quicker and more accurate interpretation when the intended meaning of the ambiguous word is congruent with the accent in which the sentence is spoken; in other words, as in Experiment 4, we predict an interaction between accent and meaning if the accent modulation on meaning access extends to sentence-level language comprehension.

It should also be noted that, apart from being able to test the accent effect in the context of sentence comprehension, Experiment 5 additionally allows for a stricter test of the immediacy of the accent modulation than Experiment 4: rather than responding to a subsequent probe that could guide further interpretation (as in Experiment 4), participants responded to the ambiguous word itself.

### 6.1 Method

#### 6.1.1 Design

The experiment adopted a 2 (accent: British vs. American) × 2 (meaning: British vs. American) design. As in previous experiments, accent was manipulated within items but between participants (to avoid accent salience) such that a participant listened only to only either British or American accented sentences. Meaning was manipulated within both items and participants. Four lists of materials were created such that different versions of each item were assigned to the lists in a Latin-square manner.

#### 6.1.2 Participants

A total of 134 British English speaking participants were recruited online from Prolific Academic, using the same eligibility criteria as in Experiment 1. Of these, 10 were excluded for reporting to have lived in the USA for more than 3 months, 4 for technical problems, and 2 for having an accuracy rate <60% for the trials. These left us with 118 participants (average age = 30.1, SD = 8.2; 57 women; 58 participants for the British accent and 60 for the American accent). All participants were compensated £1.50 for their time.

#### 6.1.3 Materials

We created target sentences for 24 ambiguous words (see Appendices A and B) selected from the pool of words whose meaning dominance we had pre-tested. For these words, the British meaning was rated as more familiar by British English speakers (British meaning: *M* = 6.25, *SD* = 0.67, range = 4.64–6.82; American meaning: *M* = 4.66, *SD* = 1.08, range = 3.03–6.59). For each item, the sentence-final ambiguous word was disambiguated towards the British meaning in one version (e.g., “The mechanic needed to repaint the whole bonnet”) but towards the American meaning in another (e.g., “The woman decided to iron her daughter’s bonnet”). All the target sentences were sensible if the intended meaning of the ambiguous word was retrieved, but were nonsensical with the other meaning. We constructed 48 filler sentences and 8 practice sentences. Twelve fillers were sensible sentences and 36 were nonsensical (e.g., “After falling off the cliff, the truck swam towards the island”). Half of the practice sentences were sensible. For the nonsense sentences, the source of the semantic anomaly varied across different positions in the sentence. Each participant heard 80 sentences in total, of which half were sensible. All the sentences were read by the same British and American speakers used for recordings in the previous experiments and recorded digitally in the same recording booth as materials for previous experiments. As in previous experiments, the two speakers attended different recording sessions to avoid accent contamination. The speech files were digitally adjusted to have comparable loudness and were edited such that they ended with the acoustic offset of the sentence-final word. There was no statistical difference in the duration of the speech files between the two speakers for either the target sets (2316 vs. 2312 ms for the British and American versions respectively, *t*(47) = 0.15, *p* = 0.884) or the practice and filler sentences (2741 vs. 2715 ms for the British and American versions respectively, *t*(55) = 1.11, *p* = 0.271).

#### 6.1.4 Procedure

The experiment was conducted online in the same manner as Experiment 4. In each trial, a fixation cross was displayed for 500 ms, followed by a spoken sentence with the fixation still on the screen. At the offset of the sentence-final word, a judgement page appeared with the text “Does NOT make sense” on the left-hand side, with the response cue “f” written beneath, and with the text “Makes sense” on the right-hand side with the response cue “j” written beneath. Participants pressed the J key with their right index finger if they thought the sentence was sensible, and the F key with their left index finger if they thought it was not. After the response, there was a blank inter-trial interval of 1 s. The whole experiment lasted for about 20 min.

### 6.2 Results

**Fig. 6:**
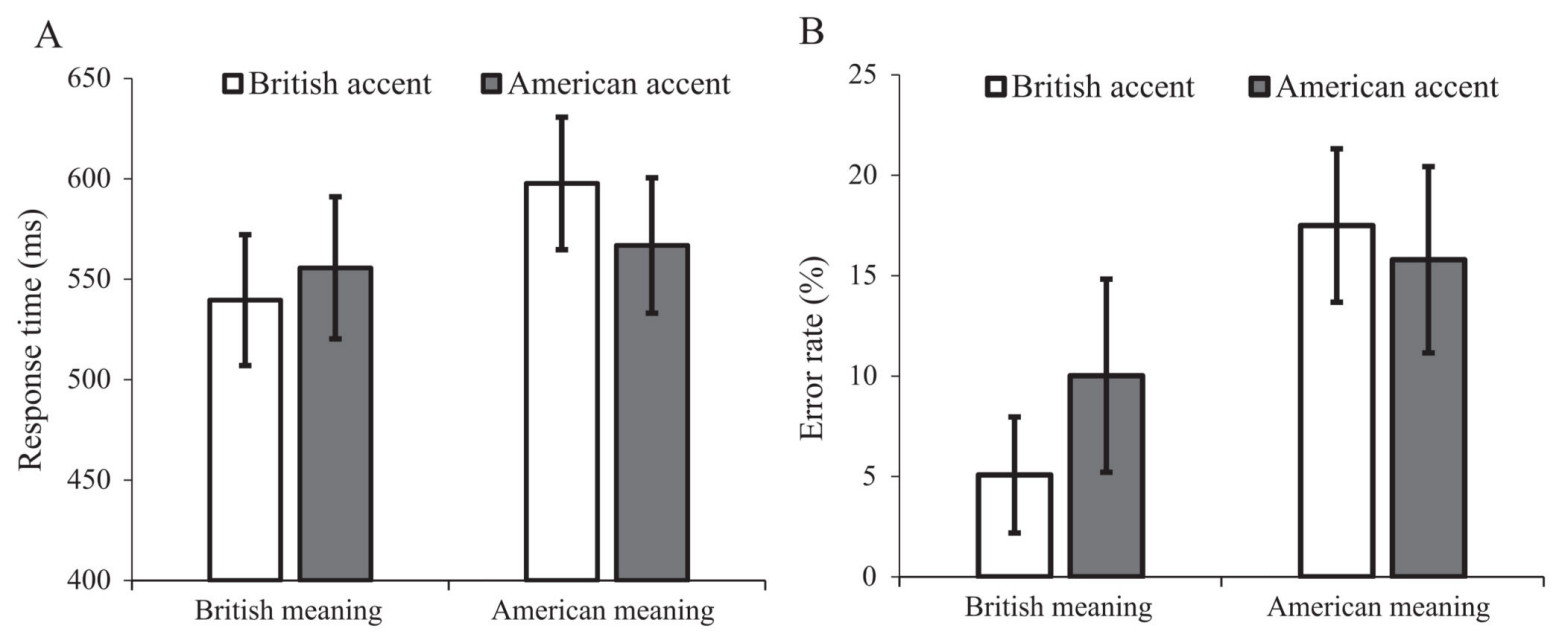
Response times (A) and error rates (B) in Experiment 5. Error bars show ±1 SE, adjusted to show within-participant effects of meaning (Morey, 2008).

For the RT analyses, we removed 352 (out of 2832) trials where participants made an incorrect response. Next, to meet the assumptions of normally-distributed residuals and homogeneity of variance, for the remaining 2480 trials, we log-transformed the RTs and then excluded RTs that were 2 SDs away from the mean for each participant, leading to the exclusion of 109 trials. For the remaining 2371 trials, we conducted linear mixed-effects modelling on the log-transformed RTs. The fixed effects included accent and meaning (both contrast-coded) and their interaction. Accent did not produce a significant main effect (*χ*^2^(1) = 0.02, *p* = 0.880). Meaning had a marginally significant main effect (*χ*^2^(1) = 3.18, *p* = 0.075), showing that responses were faster when the ambiguous word was biased towards the British than American meaning. Critically, there was also a significant interaction between accent and meaning (*χ*^2^(1) = 4.30, *p* = 0.038), with quicker responses when the intended meaning was congruent with the accent than when it was not (see Fig. 6A). Thus, this finding demonstrates that the accent modulation of on-line word meaning extends to sentence comprehension.^4^

For the error rate analysis, we included both correct and incorrect responses except for 25 very slow responses (>2500 ms) which were probably due to inattention (note that, unlike Experiment 4, trials with very quick responses were not excluded because, in this task, participants could make a valid response immediately upon the offset of the sentence-final word). An LME model with response accuracy (correct vs. incorrect responses) as the dependent variable and accent and meaning (both contrast-coded) as predictors revealed that accent had a marginally significant main effect (*β* = −0.38, *SE* = 0.23, *z* = −1.66, *p* = 0.096), with participants making slightly more errors when listening to American than British accent. Meaning had a significant main effect (*β* = −0.82, *SE* = 0.38, *z* = −2.16, *p* = 0.031): again participants made fewer errors when the sentence required a British than American-meaning interpretation for the sentence-final ambiguous word. Importantly, there was a significant accent-meaning congruency effect, as revealed by the significant interaction between accent and meaning (*β* = 1.03, *SE* = 0.30, *z* = 3.43, *p* < 0.001). This interaction indicates that participants made fewer errors when the sentence required an accent-congruent than accent-incongruent meaning for the ambiguous word.^5^

### 6.3 Discussion

In this experiment, participants were required to decide whether sentences containing ambiguous words made sense. The accuracy and speed of these responses for our critical test sentences depended on whether, and how quickly, a participant retrieved the appropriate meaning of a sentence-final ambiguous word. We showed that participants were quicker and more accurate when the sensible interpretation of the sentence required a meaning that was congruent with the accent that the sentences were spoken in. This finding suggests that speaker accent directly modulates on-line meaning retrieval, as we also have shown in Experiment 4; in addition, it showed that the accent modulation is not limited to single-word comprehension but applies to real-life language use where ambiguous words are used in sentential contexts.

## 7 General discussion

In five experiments, we found that a speaker’s accent can modulate listeners’ interpretations of ambiguous spoken words. Specifically, listeners are more likely (in word association) and quicker (in semantic relatedness judgement and sentence interpretation) to retrieve an accent-congruent meaning than an accent-incongruent meaning for an ambiguous word. For example, “bonnet” is more readily interpreted as referring to a car part when it is spoken in a British than American accent. This finding has important consequences for models of lexical processing: it demonstrates that information about the surface form of speech (i.e. accent) can influence word meaning access. This finding is in sharp contrast with the claims instantiated in many influential current models of word recognition, in which surface speech details such as accent play no direct role in meaning access. In these models, the lexical representations that are activated from speech recognition and provide the input for semantic access are abstracted away from the surface phonetic peculiarities (e.g., McClelland & Elman, 1986; Mirman, McClelland, & Holt, 2006; Norris, 1994).

In the introduction, we contrasted two theoretically distinct mechanisms whereby the surface speech details such as accent could modulate word-meaning access. The surface-detail account, which is most easily accommodated by episodic models of lexical representations, allows variants of a word with different accent properties to preferentially map onto meanings that are congruent with that particular dialect (e.g., American-accented tokens of “bonnet” are more likely to be used to refer to a hat, hence stronger links from American-accented lexical memories to the hat meaning). Critically, such a mechanism predicts that the meaning that is accessed for each instance of an ambiguous word would depend on the strength of the acoustic-phonetic accent cues in that specific speech token (and the extent to which each accented token maps preferentially onto one or other known meaning). An alternative mechanism that allows for accent modulation of meaning access is the speaker-model account; that is, speaker information is extracted from speech tokens, in parallel to the retrieval of the abstract lexical representation. Listeners can thereby make inferences about the speaker and in turn use this information to constrain their interpretation of the intended meanings of the words produced by that speaker. Several aspects of the current data are inconsistent with the former mechanism but in line with the latter proposal.

First, we tested the prediction of the surface-detail account that the strength of accent effect would be correlated with the strength of the accent cues that were present in specific word tokens. We found no support for this prediction: the magnitude of the accent effect was not modulated by either the rated (dis)similarity between a word’s British and American pronunciations (Experiment 1) or the ease with which words could be classified as having a British or American accent (Experiment 2). The accent effect was no larger for words like “bonnet” that have very different pronunciations in the two accents, compared with words like “mate” that are more similar. Perhaps more convincing than these null findings is the positive demonstration (in Experiment 2) that meaning access can be modulated by speaker accent even for neutral-accent speech tokens: the same neutral-accent tokens were interpreted differently when they were embedded within a context of either British- or American-accented words that were perceived as coming from the same speaker. Taken together, these findings support the view that listeners build up a consistent representation of a speaker’s accent across all experiences with that speaker, and use this information to modulate meaning access for all subsequent spoken words that are identified with the same speaker. That is, listening to words spoken in an American accent leads listeners to construct a model of an American English speaker and accordingly use their knowledge of American English to aid their comprehension of the all the subsequent words that the speaker produces.

One further observation in Experiment 3 confirms that this accent context is specifically tied to a model of the person speaking. Accent modulation of word meaning access applied only to spoken words and did not extend to written words intermixed with the accented speech. This finding suggests that the observed accent effects do not arise from a general form of priming (whereby all American dominant meanings are activated via a direct influence from hearing speech that signals “American-ness”) or from a general response bias (whereby, for instance, American accent in the speech prompts participants to generate more American meaning responses). If that was the case, then we should expect accent to also modulate meaning access for written words, contrary to the findings of Experiment 3.

Experiments 4 and 5 provide response time data showing that a meaning becomes available to the listener more quickly if it is congruent with the accent. This suggests that accent modulation does not simply reflect an off-line strategy in meaning choice (i.e. in hearing an American accent, consciously selecting the hat-meaning of “bonnet” rather than the car-part meaning when both meanings come to mind). Accent modulation of meaning access is still apparent in two speeded tasks (semantic relatedness and sentence coherence judgements) during which participants did not have sufficient time to engage in deliberate contemplation of which of the different meanings is more appropriate. Instead, these findings suggest that the speaker model constrains on-line meaning access by boosting accent-congruent meanings, making them more readily available for retrieval. Experiment 5 additionally showed that modelling the speaker’s dialectic background is not confined to the meta-linguistic tasks that we used to assess semantic ambiguity resolution; it also occurs in more ecological settings in which people comprehend sentences that contain ambiguous words that are used differently in different dialects.

The construction of a speaker model may happen relatively early in interacting with other speakers, perhaps in response to hearing a sufficient period of speech input to establish their dialectic identity (e.g., Witteman et al., 2014). Such a conclusion is based on the repeated (null) finding that the accent effect does not significantly increase or decrease during the course of our experiments. Once an appropriate speaker model has been established, listeners continue to use this model in interpreting incoming speech (see also Kraljic, Samuel, & Brennan, 2008, for a similar conclusion in speech perception).

It seems reasonable to propose that this speaker model will combine with other contextual cues and guide access to meaning for as long as there is no information contradicting that speaker model (e.g., introduction of a different accent), and as long as listeners believe that the speech is produced by a single person. This account then explains why the accent effect does not change as a function of the word’s accent strength: neutral-accent words in Experiment 2 do not carry conflicting accent information and are interpreted against the same speaker model as the strong-accent words, resulting in similar accent effects for the two types of words. However, in Experiment 3, listeners constructed a speaker model on the basis of the spoken words and interpreted the spoken words, but not the written words, against that speaker model (hence the lack of an accent effect for written words intermixed with spoken words). In the absence of perceptual cues (such as voice identity) to suggest that the written words come from the same person as the spoken words, participants seem to refrain from extending the speaker model to the written words.

### 7.1 Implications for models of spoken word comprehension

**Fig. 7:**
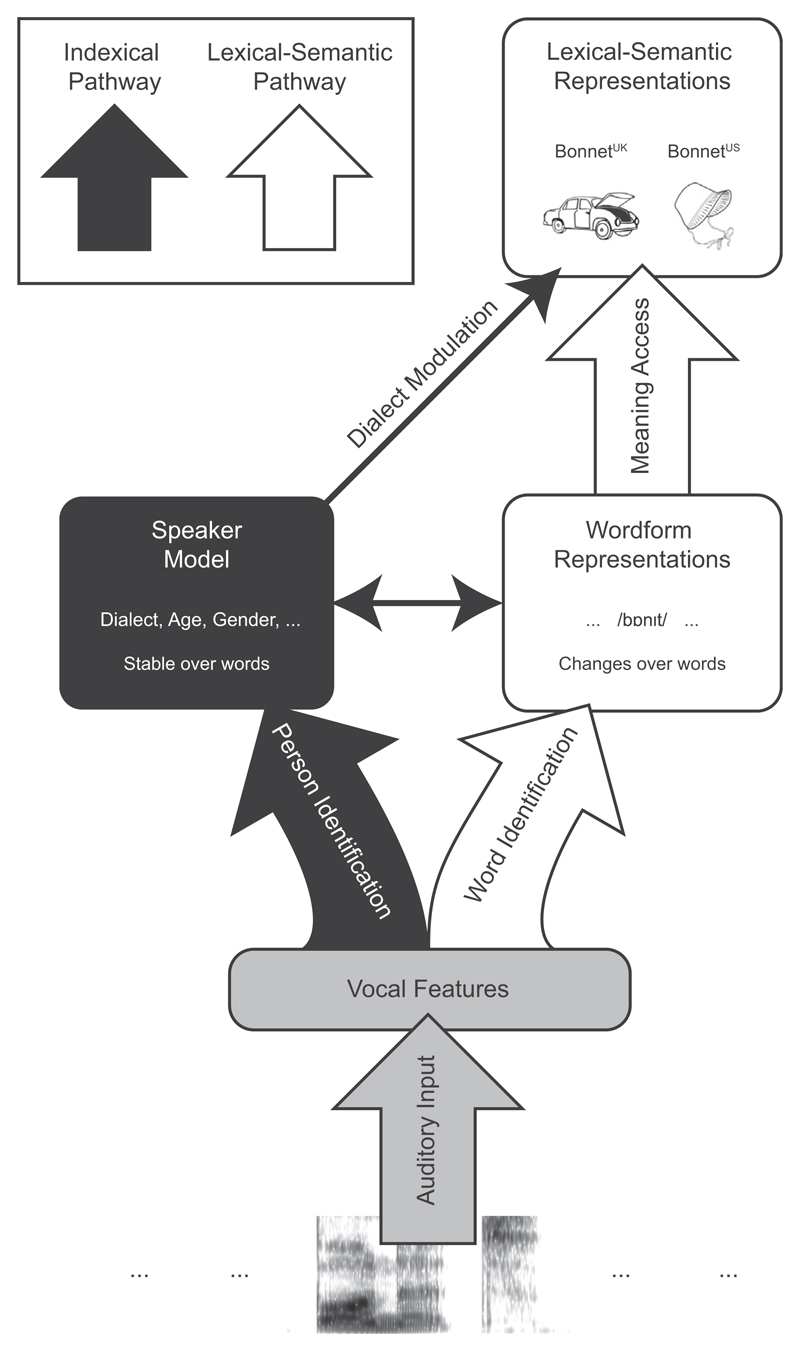
A model of spoken word comprehension which incorporates both lexical-semantic and indexical pathways. The incoming speech signal contains information concerning who is speaking (indexical information) and what they are saying (phonetic information). These information sources are processed in separate, but interacting, pathways. White (unfilled) arrows represent the lexical-semantic pathway, in which speech information is used to identify wordform representations, that in turn allow direct access to lexical-semantic representations; black (filled) arrows represent an indexical pathway, in which information in the speech signal is used to infer characteristics of the speaker, which in turn can be used to modulate lexical-semantic processing (as shown by dialect modulation in the present studies).

On the basis of the five experiments reported here we propose a model of speech processing in which listeners make use of (at least) two parallel mappings in lexical processing and meaning access: a lexical-semantic pathway that maps speech information onto phonemes, syllables and eventually lexical representations (as in standard accounts of spoken word recognition), and an indexical pathway that simultaneously and in parallel uses surface speech details to make inferences about characteristics of the person speaking (e.g., gender, age and dialectic background; see Fig. 7 for an illustration of such a model).

In the lexical-semantic pathway, phonetic information in speech is processed in order to identify the wordforms that are stored in the lexicon. These wordform representations are the primary means by which listeners access word meaning (i.e. activate lexical-semantic representations that are consistent with the sounds that they have heard). In addition, the indexical pathway extracts surface speech details that are characteristic of the person speaking rather than the words that they are saying (e.g., vocal tract length reflected in average formant frequencies, Smith, Patterson, Turner, Kawahara, & Irino, 2005; age as reflected in the harmonic-to-noise ratios, Ferrand, 2002). The dialectic identity of the speaker is established on the basis of a variety of cues, using both information about the surface features of the incoming speech, as well as information from the lexical pathway. The primary purpose of this indexical pathway is to perform speaker identification from the voice (cf. Belin et al., 2004); however, as the present experiments demonstrate, this speaker model also contributes to word meaning access.

These two pathways are unlikely to be functionally independent. For instance, listeners can use the speaker accent (e.g., British or American; information from the indexical pathway) to evaluate the appropriateness of lexical choice (e.g., “holiday” or “vacation”; information in the word-form representations, Martin et al., 2016). Similarly, information about the accent that a speaker uses can also guide wordform identification (i.e. perceptual learning of unfamiliar accents leads to faster and more accurate word recognition, cf. Clarke & Garrett, 2004; Mitterer & McQueen, 2009). Conversely, there is evidence that one’s social perception of another person (e.g., intellectual competence or political allegiance) can be influenced by that person’s lexical choices (Bradac, Konsky, & Davies, 1976; Porter, Rheinschmidt-Same, & Richeson, 2015). These and other phenomena lead us to believe that speaker and word identification operate from a shared representation of vocal features and are mutually constraining (indicated by the double-ended arrow between ‘Wordform Representations’ and ‘Speaker Model’ in Fig. 7). The specific details of these functional interactions remain to be specified in future work.

Importantly, the present work has shown a very specific form of interaction between lexical-semantic and indexical pathways: that dialect information in the indexical pathway is used to constrain meaning access. The speaker model will, in the case of semantically ambiguous wordforms (e.g., “bonnet”), bias lexical-semantic processing towards lexical-semantic representations that are consistent with both the wordform and additional knowledge of the speaker’s dialectic identity. For example, in the case of a British listener hearing “bonnet” spoken in an American accent, knowledge of speaker dialect will increase the availability of the subordinate *hat* meaning. In the absence of a constraining context, (Experiments 1–3), this will make it more likely that the final settled state will correspond to this meaning. This constraint satisfaction account explains why accent cues did not completely overturn listeners’ overall dominance biases (e.g., for our British participants, the British dominant meaning was, on average, more likely to be retrieved even when a word was heard in an American accent). Instead, accent cues resulted in a modulatory effect, facilitating access to the meaning that is congruent with the inferred dialect of the speaker. In the presence of a prior constraining context (Experiment 5) or a subsequent cue (Experiment 4) that mandates access to a specific meaning, access to accent-congruent meanings will be faster and more accurate. While the source of this dialect modulation is distinct from other forms of contextual or linguistic bias (prior knowledge, meaning frequency, etc.), we suggest that similar constraint satisfaction processes operate to integrate information from a range of cues to allow the listener to select the most likely meaning for the current utterance.

This distinction between the lexical-semantic and indexical pathways is closely aligned with recent proposals concerning spoken language processing (Belin, Bestelmeyer, Latinus, & Watson, 2011; Belin et al., 2004; Martin et al., 2016; Sumner et al., 2014; Van Berkum et al., 2008) and in broad agreement with the proposal that speech perception involves parallel representations of indexical and linguistic information (e.g., Magnuson & Nusbaum, 2007; Pierrehumbert, 2016; Zhang & Chen, 2016). For instance, based on a range of cognitive and neuro-cognitive studies, Belin et al. (2004, 2011) propose that speech input is processed by three parallel processing pathways that respectively perform analysis of (i) speech information, (ii) vocal identity, and (iii) vocal affective information. The key difference between our account and those proposed by Belin and colleagues is that the speaker model not only helps determine *who* is speaking, but can also influence the processes by which listeners determine which *meaning* was intended for a given word.

In terms of spoken word recognition, our proposed model does *not* require that we abandon the influential abstractionist approach to word recognition in order to accommodate the effect of surface details on word recognition and meaning access. Under our proposal, *all* instances of a particular spoken word (regardless of variation in accent or other acoustic properties) could initially map onto a shared abstract/phonological lexical representation, in the same fashion as instantiated in many abstractionist models of spoken word recognition (e.g., McClelland & Elman, 1986; Norris, 1994). By assuming that surface speech details are discarded during the word recognition process, these models are consistent with the finding that accent cues available in individual speech tokens do not appear to have a strong/reliable effect on modulating meaning access. Indeed, our data suggest that accent cues are not used to directly identify lexical representations and access word meanings. In particular, the finding from Experiment 2 that speech morphs devoid of accent cues can bias meaning access just as strongly as neighbouring accented speech tokens suggests that it is the speaker model rather than token-specific accent cues that modulates meaning access.

An important feature of our proposed model is that it appears to require a level of representation in which the different meanings of an ambiguous word correspond to distinct representations (‘Lexical-Semantic Representations’ in Fig. 7). Without these representations it is not clear how the dialect modulation effect could arise. This effect cannot arise at an earlier stage (i.e. during word identification) as the different meanings of the ambiguous words are accessed via the same (abstractionist) wordform representation. But equally, dialect modulation cannot arise at a purely semantic level: there is no reason to think that hearing a British or American accent would favour clusters of *car-part* or *hat* meanings per se; only when speakers of these dialects use the word “bonnet” should car-part or hat representations be differentially accessed. In other words, the observed accent effect *must* be viewed as a modulation of lexical-semantic representations, not of purely semantic features.

This raises important questions about the nature of the lexical-semantic representations that are modulated by dialect (and potentially other person-based) information. One possibility is that these representations are (as depicted in Fig. 7) localist ‘word-meaning nodes’ – one node per lexical meaning. Such an account could accommodate our results, for example by assuming that activation of a particular dialect (within the speaker model) would boost the activation of those word meanings that were more strongly associated with that dialect. However, this view that the lexical-semantic representation of individual word meanings can be captured by individual localist nodes seems somewhat unlikely. While this approach may be plausible for representing words with a small number of distinct, semantically unrelated meanings, they are less parsimonious when considering the more common phenomenon of polysemy: that most words have numerous semantically related word senses (Rodd, Gaskell, & Marslen-Wilson, 2002, 2004). In this case it becomes somewhat arbitrary whether or not alternative senses are considered sufficiently distinct that they require additional localist lexical-semantic nodes. It is not clear how the semantic richness of polysemous words such as “run” and “line” that have very large numbers of related word senses can be explained within a localist framework.

Instead, we favour distributed connectionist models of lexical semantics in which the individual meanings of familiar words are represented as stable states within a complex multi-dimensional lexical-semantic space (e.g., Rodd et al., 2004). Under this distributed connectionist view, the multiple semantically-related senses of polysemous words correspond to different stable states that cluster close together within this semantic space, while the different unrelated meanings of homonyms correspond to more distant stable states. Rodd et al. (2004) suggested that when a semantically ambiguous word is encountered it is initially mapped onto a ‘blend state’ within this semantic space that corresponds to a (meaningless) combination of its different meanings, but then an active settling process allows the activation to resolve towards a stable coherent representation that corresponds to just one of these meanings. Within this framework, the dialect modulation effect could operate in the same manner as other contextual biases during this settling process by (i) increasing the likelihood that the network settles into the accent-congruent meaning and (ii) decreasing the time taken to reach this final settled state. But it remains to be shown using detailed computational simulations that the results from the current paper can be simulated by connectionist models of this type. The current data do not speak directly to the nature of these lexical-semantic representations and indeed we see the debate between localist vs. distributed representations of word meanings as an orthogonal implementation issue independent of the functional questions that we addressed here concerning how the speaker model modulates meaning activations.

More broadly, this research emphasises the need to integrate models of meaning access into a more general framework that considers both spoken word recognition and person identification (e.g., Belin et al., 2004, 2011). We speculate that accent is just one of many informative cues (e.g., age, gender) that are routinely identified by listeners in order to facilitate language comprehension. Identifying how a particular speaker is likely to use specific words in the language can potentially help alleviate the additional cognitive load that is associated with accessing strongly subordinate meanings (see Johnsrude & Rodd, 2015; Rodd et al., 2010), and reduce the likelihood that such ambiguous words, which are ubiquitous in natural language, will be misinterpreted. Further experiments will be required to demonstrate what other cues can be effectively used to infer meanings intended by a speaker (or writer). One intriguing possibility, for instance, is that listeners keep track of how individual familiar speakers use words, and that this distributional information allows them to make highly tuned predictions for word meanings in the speech of those people that they know best (their friends and family). This might in turn require highly detailed speaker models linked to episodic memories of conversations with individual speakers, their accents, and word usage.

### 7.2 Implications for spoken communication

Our conclusion that listeners use speaker-related details to construct a speaker model to guide word interpretation is broadly consistent with the proposals that spoken communication involves alignment at different linguistic levels between interlocutors (Pickering & Garrod, 2004). Inter-interlocutor linguistic alignment has been found to occur automatically at a variety of levels. For instance, interlocutors gradually shift their accent, speech rate and articulation towards each other during conversation (Giles, Coupland, & Coupland, 1991; Pardo, 2006). They automatically converge on lexical choices during object naming tasks (e.g., whether to use *couch* or *sofa* to name a piece of furniture; Brennan & Clark, 1996; Clark & Wilkes-Gibbs, 1986). They are also found to repeat each other’s syntactic structure (e.g., actives vs. passives) when they take turns to describe depicted events (Branigan, Pickering, & Cleland, 2000; Cai, Pickering, & Branigan, 2012). Some other forms of alignment may reflect more strategic efforts to take a shared perspective with a conversational partner. For instance, interlocutors tend to gradually use the same expressions for identifying spatial positions (Garrod & Anderson, 1987) and they also assess whether something is on common ground with their conversational partner when they comprehend descriptions of objects in a scene (Hanna et al., 2003; Keysar et al., 2000).

In this context, our study supports these previous findings by suggesting that listeners make interlocutor-specific inferences at different linguistic levels during language comprehension. Martin et al. (2016) demonstrated that listeners have expectations of typical lexical expressions by the speaker on the basis of the speaker’s accent (e.g., expecting an American English speaker to use “vacation” rather than “holiday”). Van Berkum et al. (2008) showed that listeners can quickly detect the pragmatic infelicitousness if, for instance, the utterance “I have a large tattoo on my back” is spoken in an upper-class accent, suggesting that listeners make speaker-specific pragmatic inferences in on-line language comprehension. Listeners also make interlocutor-specific semantic interpretations. For instance, listeners use the speaker’s spatial location when interpreting instructions from the speaker to move objects around (Ryskin, Wang, & Brown-Schmidt, 2016); they also assess the interlocutor’s knowledge (what is known/hidden to the interlocutor) in interpreting utterances from the interlocutor (Hanna et al., 2003; Keysar et al., 2000). Finally, there is some evidence that listeners may develop speaker-specific conceptual references for words, at least temporarily, in dialogue settings where different speakers may use different terms to describe the same referent (Brown-Schmidt, 2009).

Our study further adds to this literature by showing that listeners use the dialectic identity of the speaker (established via accent cues) to interpret word meanings. In contrast to earlier findings concerning interlocutor-specific inferences in interactive situations in which listeners comprehend sentences in extended dialogues, our findings suggest that listeners are able to utilise cues about the speaker despite the speaker not being physically present (see also Martin et al., 2016; Van Berkum et al., 2008; cf. Brown-Schmidt, 2009) and despite the linguistic materials being (for Experiments 1–4) single spoken words heard in isolation. Even very simple spoken word recognition tasks are hence influenced by processes that guide interpretation based on knowledge of the linguistic background of a speaker.^6^

## 8 Conclusion

In conclusion, the current study showed that listeners use accent information to establish the dialectic background of the speaker and use their knowledge of that linguistic variety to guide access to the meanings of the words uttered by the speaker. In doing so, they map accented speech onto abstract wordform representations and, at the same time, extract speaker-related surface speech details (e.g., accent) to make inferences about the speaker (e.g., dialectic background). Both the wordforms and the speaker model then feedforward to constrain the meaning intended by the speaker.

## Supplementary Material

Appendices

## Footnotes

1 There is in fact no direct attribution of this quote to Shaw, though the 1951 Treasury of Humorous Quotations (Esar & Bentley) quoted him saying “‘England and America are two countries separated by the same language” but did not give the source.

2 Though the significant interaction between speaker accent and probed meaning clearly demonstrates accent modulation of on-line meaning access, one may ask whether there were significant simple effects of accent when the two meanings were taken separately. Visual inspection of Fig. 5A suggests that the American accent led to longer RTs than the British accent when the British meaning was probed but the reverse when the American meaning was probed; however, the simple effect of accent turned out to be non-significant in both cases (British meaning: *χ*^2^(1) = 2.09, *p* = 0.148; American meaning: *χ*^2^(1) = 0.187, *p* = 0.666). It should be noted that, as accent was manipulated between participants, analyses comparing two accents involved between-participant comparisons based on half of the data (i.e. comparing RTs to of one the two meanings between the British accent participant group and another group of participants hearing an American accent); therefore, these simple-effect analyses were underpowered relative to the main analysis. We believe that the more theoretically constraining finding comes from the significant accent-by-meaning interaction which clearly demonstrates an accent-meaning congruency effect, even in the absence of reliable effects in between-subject comparisons based on partial data.

3 Fig. 5B suggests that the American accent led to more errors than the British accent for the British meanings, but with the reverse pattern for the American meanings. The accent effect was significant within the British meaning conditions (*β* = −0.62, *SE* = 0.23, *z* = −2.69, *p* = 0.007) but not within the American meaning conditions (*β* = 0.12, *SE* = 0.18, *z* = 0.65, *p* = 0.517). However, as noted before, these simple effect subset analyses were underpowered as a result of the between-participant comparisons of accent groups based on partial data within each meaning condition.

4 By-meaning separate analyses revealed no significant simple effect of accent for either the British meaning: *χ*^2^(1) = 0.42, *p* = 0.516) or the American meaning (*χ*^2^(1) = 0.38, *p* = 0.535), though again it should be noted that these analyses were likely to be underpowered for reasons mentioned earlier.

5 Fig. 6B suggested more errors for the American than British accent when the ambiguous word was biased towards its British meaning, as indeed supported by the statistical analysis (*β* = −0.86, *SE* = 0.25, *z* = −345, *p* < 0.001) but fewer errors for the American than British accent when the ambiguous word was biased towards its American meaning, though this was not borne out in the statistical analysis (*β* = 0.12, *SE* = 0.25, *z* = 0.46, *p* = 0.647). However, it is likely that these separate analyses were underpowered.

6 An anonymous reviewer suggested that there may be an interesting parallel between the ambiguous words we investigated here and interlingual homographs, or “false cognates”, in bilingual lexicons (e.g., Beauvillain & Grainger, 1987; Dijkstra, Grainger, & Van Heuven, 1999). Our experiments have used words like “bonnet” that have different dominant meanings in different dialects despite being similar in their spoken form and identical in their written form; interlingual homographs such as “room” in English and “room” in Dutch (meaning “cream”) similarly have different and unrelated meanings between languages despite having similar spoken and identical written forms. We have shown that listeners use dialectic information (signalled by the accent of the speaker) to assist their understanding of ambiguous words. Whether the processing of interlingual homographs uses a similar mechanism to interpret (written) interlingual homographs remains a question for future investigation.
