# Supplementary material for: Accent modulates access to word meaning: Evidence for a speaker-model account of spoken word recognition
Source: Cogn Psychol. Author manuscript; Available in PMC 2019 Jun 27. (PMC6597358; doi:10.1016/j.cogpsych.2017.08.003)
Supplement: Appendices [file EMS83488-supplement-Appendices.pdf]

Appendix A. Stimuli used in Experiments 1–4

The British/American meaning definitions were used in the verification task. The numbers in brackets in the first column (i.e. "Experiment #") refer to the name of experiments where a word was used. The British/American meaning probes were used only in Experiment 4. Words for Experiment 5 were used in a sentence in the final position (see Appendix B). Phonetic similarity was measured for items used in Experiment 1 and accent discriminability score for target words used in Experiment 2.

Target word (Experiment #)	British meaning (familiarity ratings by British and American English speakers respectively)	American meaning (familiarity ratings by British and American English speakers respectively)	British/American meaning probe	Phonetic similarity (UK/US participants)	Accent discriminability
BANGS (1,3,4,5)	loud, abrupt noises. (6.3, 6.1)	part of a hairstyle above the face. (3.3, 6.4)	LOUD/HAIR	4.8/3.5	
BLINKERS (2,4,5)	leather flaps on a bridle to restrict a horse's lateral vision. (4.6, 2.2)	lights on a car that indicate the direction about to be taken. (3.4, 6.2)	HORSE/CAR		0.94
BONNET (2,3,4,5)	the hinged metal canopy covering the engine of a motor vehicle. (5.4, 1.9)	a woman's or child's hat tied under the chin. (4.2, 4.9)	CAR/HAT		0.94
BRACKETS (4)	form of sentence punctuation. (6.2, 5.2)	supports for shelves. (6.1, 5.5)	COMMA/SHELF		
CHEMIST (2,5)	a place to buy medicinal drugs. (6.8, 2.8)	a person engaged in chemical research or experiments. (5.9, 6.0)			0.89
CHIPS (1,3)	fried pieces of potatoes served hot. (6.4, 3.4)	thinly cut slices of fried potato usually served cold as a snack. (4.4, 6.2)		3.4/2.4	
COACH (2,4,5)	bus, usually used for longer journeys. (6.6, 3.9)	sports trainer. (6.1, 6.3)	TRAVEL/PLAYER		0.74
COMPREHENSIVE (2)	a school secondary level in which children of all abilities are educated. (6.3, 2.6)	general term to describe something that includes all or many possible components. (6.2, 5.9)			
CRICKET (2,4,5)	bat-and-ball sport. (6.7, 4.1)	insect, similar to a grasshopper. (5.9, 6.3)	SPORT/INSECT		0.89
CRISP (1,4)	thinly cut slices of potato usually served cold as a snack. (6.2, 3.4)	firm, dry and brittle. (4.9, 5.6)		3.1/2.3	
DRAPE (2)	to hang limply. (5.3, 4.7)	curtain. (4.3, 5.9)			0.84
DUMMY (2,3,4,5)	something that sooths infants. (5.3, 1.2)	a fool or idiotic person. (5, 5.8)	BABY/STUPID		0.71
FALL (1,3,4,5)	to trip over something. (6.6, 6.4)	season of the year before winter. (4.0, 6.6)	INJURY/WINTER	5/4.4	
FLANNEL (2)	cloth used for washing. (6.5, 2.3)	type of fabric/material. (5.8, 6.1)			0.87
FLAT (1,2,3,4)	a place in which to live, usually part of a larger building. (6.5, 4.6)	smooth and level. (6.4, 6.6)	HOUSE/SMOOTH	3.9/2.4	0.88
FOOTBALL (1)	a sport played with a round ball, where the objective is to kick it into a netted goal. (6.9, 4.2)	a sport played with an oval ball, where the objective is to score a touchdown. (4.3, 6.6)		5.1/4.1	

(continued on next page)

Appendix A. (*continued*)

Target word (Experiment #)	British meaning (familiarity ratings by British and American English speakers respectively)	American meaning (familiarity ratings by British and American English speakers respectively)	British/American meaning probe	Phonetic similarity (UK/US participants)	Accent discrimin-ability
GARAGE (5)	place to buy petrol (6.4, 3.2)	building serving as public parking facility (5.4, 6.2)			
GAS (1,2,3,5)	a gaseous form of a substance. (6.5, 6.3)	a fuel for motor vehicles. (4.2, 6.8)		3.2/1.6	0.83
GUTTED (1,3,4,5)	very disappointed. (6.6, 2.4)	having had its internal organs removed prior to cooking. (4.4, 5)	UPSET/FISH	6.1/3.9	
HAMPER (2,4,5)	large basket used for food, especially for picnics. (6.3, 3.0)	basket for clothes that need washing. (4.7, 6.5)	FOOD/ LAUNDRY		0.93
JELLY (1,2,3)	dessert in which a gelatine liquid is cooled and sets. (5.9, 3.4)	a type of spread, often used on bread. (3.4, 6.5)		3.3/2.5	0.87
JUMPER (1,2,3,4,5)	a long sleeved, woollen top. (6.8, 3.6)	someone that jumps. (3.8, 4.8)	CLOTHING/ ATHLETE	3.4/3.3	0.93
LIFT (1,2,3)	machine used to ascend and descend a building. (6.6, 4.0)	to raise something. (6.3, 6.4)		3.5/2.9	0.86
MAJOR (1,3)	serious or important. (6.2, 6.1)	principle field of study of a student at a university. (4.0, 6.8)		3.1/2.9	
MATE (1,2,3,4)	friend or chum. (6.8, 4.1)	nature reference, to have intercourse with another. (5.0, 5.7)	BUDDY/ REPRODUCE	1.8/1.9	0.79
NICKED (2,3,4,5)	arrested or detained by police. (4.9, 1.6)	scratched or indented creating a mark. (3.8, 5.9)	ARRESTED/ CUT		0.79
NICKEL (1,2,5)	an element, type of metal. (4.9, 5.8)	a coin worth five cents. (3.0, 6.7)		2.7/2.4	0.87
PANTS (1,2,3)	type of underwear. (6.4, 2.1)	an outer garment covering the body from the waist to the ankles. (3.9, 7)		5.3/4.1	0.88
PLASTER (1,2,3,5)	sticky material that is used to cover an open wound. (6.3, 2.8)	a substance used to coat walls. (5.4, 5.9)		5.4/4	0.88
QUARTER (1,5)	a fraction when 1 has been divided into four. (6.5, 6.1)	a coin worth 25 cents. (3.7, 6.8)		5.6/4.5	
RECEPTION (4)	area where hotel guests/visitors are greeted. (6.3, 4.9)	the quality of phone/radio/ television signal. (6.0, 6.3)	HOTEL/ SIGNAL		
RETAINER (4)	money paid to retain services of a person. (5.1, 4.9)	device for straightening teeth. (4.4, 5.2)	DEPOSIT/ TOOTH		
SHATTERED (1,3,4,5)	very tired. (6.6, 2.2)	broken or smashed into many pieces. (5.9, 6.5)	TIRED/ BROKEN	5.5/4.2	
SURGERY (2,5)	place where doctors/physicians/dentists practice. (6.7, 3.5)	act of performing a medical operation. (6.6, 6.3)			0.93
SWEDE (2)	type of root vegetable. (5.5, 1.1)	someone from Sweden. (5.3, 5.4)			0.78
TIN (4)	airtight container often used to store food. (6.7, 4.8)	silvery-white metal. (5.9, 5.9)	CAN/ COPPER		
TRAINER (1,2,3,5)	type of shoe, often worn in sports and when exercising. (6.8, 4.1)	a person who trains and helps someone achieve certain goals. (5.8, 6.4)		3.4/3.1	0.90
TROLLEY (2,5)	lightweight vehicle with wheels used during shopping. (6.7, 2.8)	tram/streetcar that runs on tracks and is powered by an electric cable. (3.6, 5.3)			0.88
TUBE (1,2,3,4,5)	an underground train, used as a means of transport. (6.8, 3.6)	type of hollow cylinder. (5.6, 6.0)	TRANSPORT/ PIPE	5.7/4.1	0.88
TWISTER (2,4,5)	game involving placing hands/feet on coloured spots. (6.4, 5.2)	tornado. (4.9, 5.9)	GAME/ WIND		0.94
VEST (1,2)	sleeveless undergarment worn for extra warmth. (6.0, 4.6)	formal sleeveless clothing, worn as part of a suit. (4.0, 5.9)		2.7/2.2	0.78
ZIP (1,5)	something that fastens two pieces of material together, often on clothes. (6.3, 4.5)	zero or nothing. (3.1, 5.0)		2.4/1.9	0.76
ZIP (2)	something that fastens two pieces of material together, often on clothes. (6.3, 2.6)	postal code. (4.3, 6.7)		2.4/1.9	0.76

Appendix B. Target sentences used in Experiment 5

Each of the 24 target sentences had a British meaning version (a) and an American meaning version (b). The target ambiguous word (see also Appendix A) is the last word in each sentence.

1a	Mary was startled because of the unexpected bangs.
1b	Mary was shy because of her new bangs.
2a	She thought the injury was due to the fall.
2b	She thought the holidays were in the fall.
3a	The woman was a keen cook and preferred to use gas.
3b	The woman wanted to drive to her friend and needed to buy gas.
4a	The girl failed her exam and was gutted.
4b	The fish were delivered to the restaurant and immediately gutted.
5a	After the class, he decided to change his jumper.
5b	After the competition, she went to congratulate the jumper.
6a	The family were excited about playing twister.
6b	The villagers were warned about the twister.
7a	The scientist tested several materials, including nickel.
7b	The woman dropped several items including a nickel.
8a	The woman opened her bag and took out a plaster.
8b	The house was old and needed new plaster.
9a	The pizza was all in quarters.
9b	The change was all in quarters.
10a	The schoolboy was shattered.
10b	The plate was shattered.
11a	The boy ripped his new trainers.
11b	The team spoke to different trainers.
12a	The tourists were coming out of the tube.
12b	The sweets were coming out of the tube.
13a	She tried to close the bag but broke the zip.
13b	She tried to collect for charity but got zip.
14a	The mechanic needed to repaint the whole bonnet.
14b	The woman decided to iron her daughter's bonnet.
15a	The woman didn't think children should need a dummy.
15b	The man didn't think people should be called a dummy.
16a	After the fight John's brother was nicked.
16b	During the fight John's face was nicked.
17a	Horses are more likely to win if they wear blinkers.
17b	Drivers are less likely to crash if they use blinkers.
18a	After seeing the doctor the woman went into a chemist.
18b	After publishing her results the woman became a famous chemist.
19a	The students got onto the coach.
19b	The students obeyed the coach.
20a	John's favourite activity was cricket.
20b	John's unusual pet was a cricket.
21a	They sold surprisingly good coffee at the local garage.
21b	They stored a surprising amount in their own garage.
22a	They put the beer and meat in the hamper.
22b	She put the socks and shirts in the hamper.
23a	Mary had an appointment at her local surgery.
23b	Mary was really scared before the important surgery.
24a	The woman went into the shop and found a trolley.
24b	The woman went into the city and boarded a trolley.
